# Somatic PMK-1/p38 signaling links environmental stress to germ cell apoptosis and heritable euploidy

**DOI:** 10.1038/s41467-022-28225-8

**Published:** 2022-02-04

**Authors:** Najmeh Soltanmohammadi, Siyao Wang, Björn Schumacher

**Affiliations:** 1 Institute for Genome Stability in Ageing and Disease, Medical Faculty, University Hospital and University of Cologne, Joseph-Stelzmann-Str. 26, 50931 Cologne, Germany; 2 Cologne Excellence Cluster for Cellular Stress Responses in Ageing-Associated Diseases (CECAD), Center for Molecular Medicine Cologne (CMMC), University of Cologne, Joseph-Stelzmann-Str. 26, 50931 Cologne, Germany

**Keywords:** Apoptosis, Chromosomes, Genomic instability, Genomic instability, DNA damage and repair

## Abstract

Inheritance of stable and euploid genomes is a prerequisite for species maintenance. The DNA damage response in germ cells controls the integrity of heritable genomes. Whether and how somatic stress responses impact the quality control of germline genomes has remained unclear. Here, we show that PMK-1/p38-mediated stress signaling in intestinal cells is required for germ cell apoptosis amid ionizing radiation (IR)-induced or meiotic DNA double strand breaks (DSBs) in *C. elegans*. We demonstrate that intestinal PMK-1/p38 signaling regulates the germ cell death in response to environmental stress. The PMK-1/p38 target SYSM-1 is secreted from the intestine into the germline to trigger apoptosis of meiotic pachytene cells. Compromised PMK-1/p38 signaling in intestinal cells leads to stress-induced aneuploidy in the consequent generation. Our data suggest that somatic stress surveillance controls heritable genome integrity and euploidy.

## Abstract (web summary)

Here the authors show that elimination of germ cells carrying damaged genomes is regulated by intestinal stress signalling in nematodes. Failure of the intestinal signalling results in stress-induced aneuploidy indicating that environmental stress impacts inheritance.

## Introduction

The maintenance of genomic integrity in germ cells is a prerequisite for fertility and species maintenance. Cells invoke the DNA damage responses (DDR) to induce cell cycle arrest to allow time for the DNA repair machineries to remove the damage or to eliminate genomically compromised cells through apoptosis^1^. In the germ cells, integration of efficient meiotic checkpoint signaling with the canonical DDR ensures the maintenance of numerical chromosomal composition, which is crucial for successful reproduction and prevention of birth defects^2^. In *Caenorhabditis elegans*, the evolutionary highly conserved DDR functions in the germline, which is the only proliferative tissue in adult worms^3^. Upon ionizing radiation (IR), the DDR induces cell cycle arrest in mitotic cells in the distal germ stem cell compartment, while late meiotic pachytene cells carrying DSBs succumb to apoptosis^3^. In contrast to germ cells, somatic tissues are highly radioresistant^3–5^. In the germline, checkpoint signaling is initiated when the conserved 9-1-1 complex proteins including MRT-2 (*RAD1*), HPR-9 (*RAD9*), and HUS-1 (*HUS1*) recognize DNA double-strand breaks (DSBs)^6,7^. DNA damage-induced germ cell apoptosis is triggered by the activation of the *C. elegans* p53 homolog, CEP-1, which transcriptionally induces the expression of the BH-3 only domain genes *egl-1* and *ced-13*^6,8–10^. EGL-1 and CED-13 then bind to the Bcl-2 homolog CED-9 consequently unleashing the Apaf1-like CED-4 to activate the apoptosis executioner caspase CED-3^6,10,11^.

Similar to IR-induced DNA damage, germ cell apoptosis is triggered when unresolved DSBs result from meiotic recombination failure that occurs when homologous chromosomes are unpaired and/or unsynapsed resulting in incomplete crossovers (COs)^12^. This apoptotic response is evident in mutants, such as *syp-2*, that are incapable of forming proper synaptonemal complexes (SCs). In such mutants, homologous chromosomes fail to align tightly during the pachytene phase of meiosis resulting in unresolved, persistent meiotic DSBs^13–16^.

In *C. elegans* as well as in mammals, mitogen-activated protein kinase (MAPK) signaling pathways play a critical role in the regulation of apoptosis^17–20^. It has been suggested that, while ERK1/2 MAPK predominantly facilitates cell survival, the JNK and p38 MAPKs, both characterized as stress-responsive kinases, promote apoptosis. Recent studies, however, have challenged this simplistic view and suggested a more context and cell type-specific impact of the various MAPK pathways in the regulation of apoptosis in mammals^21^. It was previously reported that the ERK MAPK homolog, MPK-1, is required in *C. elegans* to promote germ cell apoptosis in response to DNA damage^18^. Moreover, we have shown that MPK-1 induces the expression of immune genes and regulates germline DNA damage-induced systemic stress resistance (GDISR)^22^. Both findings suggest that the role of MAPKs in modulating DDRs is evolutionary conserved. The p38 homolog PMK-1 is the terminal MAPK of a stress and also immune responsive signaling pathway whose activity seems to be restricted to somatic tissues, particularly the intestine as well as neurons^23–26^. PMK-1 was shown to regulate the innate immune response to pathogen infection^27^ and is required to induce somatic stress resistance in response to intestinal infection by *Pseudomonas aeruginosa*^22^. In addition, the induction of germline apoptosis in response to *Salmonella enterica* infection is dependent on PMK-1^20^. While PMK-1 was demonstrated to be required in response to DNA damage for induction of germ cell apoptosis^28^, it has remained unclear how PMK-1/p38 impacts the regulation of germ cell apoptosis.

Here, we report that the PMK-1/p38 MAPK stress-responsive pathway in intestinal cells is required to induce germ cell apoptosis in response to IR- and persistent meiotic DNA damage in *C. elegans*. We determine that PMK-1/p38 acts upstream of CED-9/Bcl-2 for the regulation of DNA damage-induced germ cell apoptosis. Intriguingly, intestinal PMK-1/p38 regulates the synergistic effect of heat stress in elevating DNA damage-induced germ cell apoptosis, suggesting an intestinal stress surveillance mechanism that regulates the DDR in the germline. We demonstrate that the PMK-1 target SYSM-1 is secreted from the intestine and mediates the non-cell-autonomous control of DNA damage-induced germ cell apoptosis. Finally, intestinal PMK-1/p38 was crucial to ensure the accurate inheritance of homologous X chromosomes upon heat stress or DNA damage. We propose that PMK-1/p38-mediated somatic stress surveillance regulates meiotic chromosomal quality control thus promoting heritable diploidy. Taken together, our data establish that somatic stress signaling regulates chromosome inheritance.

## Results

### PMK-1 regulates germline apoptosis in response to DNA damage

**Fig. 1:**
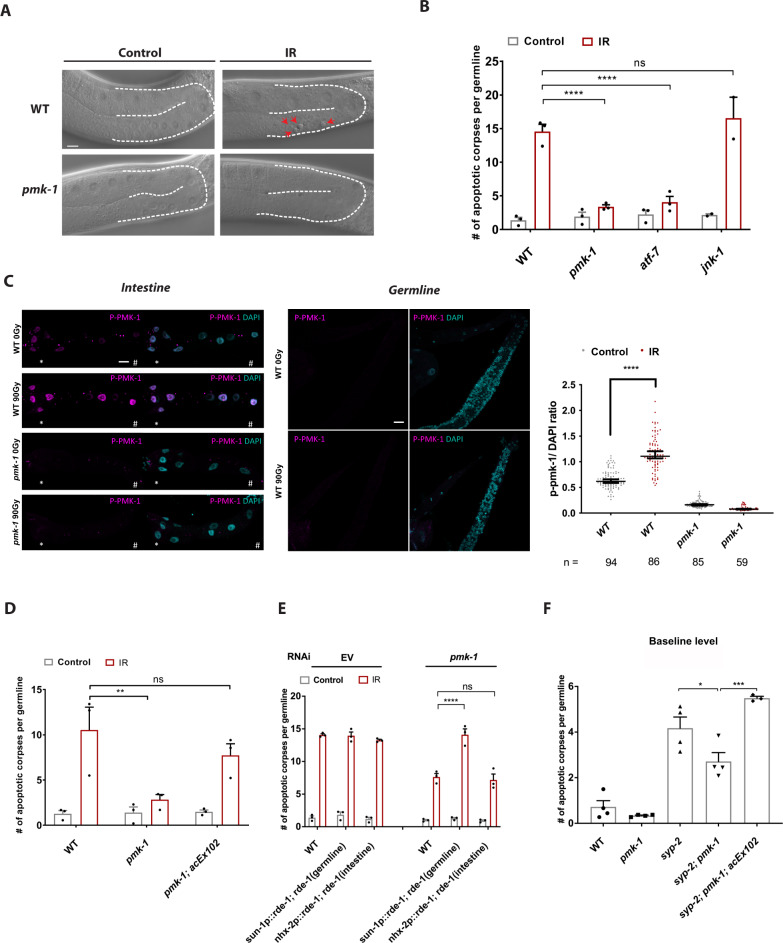
Intestinal PMK-1/p38 regulates germ cell apoptosis in response to DNA damage through a non-cell-autonomous mechanism. **A** Representative DIC images of IR and untreated WT and *pmk-1(km25)* mutant germlines taken 6 h post-IR irradiation. WT worms displayed increased numbers of apoptotic corpses in the gonad loop of the germline (highlighted by red arrows). The scale bar corresponds to 20 μm. **B** Quantification of apoptotic corpses per germline revealed suppressed levels of apoptosis in *pmk-1(km25)* and *atf-7(qd22*,*qd130)* mutant animals, whereas the apoptosis levels in *jnk-1(gk7)* mutant animals was comparable to those in WT worms in response to IR. (Two-way ANOVA with Tukey’s multiple comparison test. *WT(IR)*: *pmk-1(IR)* ****p < 0.0001, *WT(IR)*:*atf-7(IR)* ****p < 0.0001, *WT(IR)*:*jnk-1(IR)* ns: *p* = 0.8661). **C** Immunofluorescence staining of p-PMK-1 (violet) in dissected intestine and germline of the indicated strains with or without IR. Merged images with DAPI (turquoise) were shown. *Indicates anterior side; ^#^indicates posterior side. Scale bar = 20 μm. Right panel shows the quantification of p-PMK-1 signal intensity from **C**. DAPI was used for normalization. Sample sizes in C are indicated. Data are presented as median with 95% CI (Two-way ANOVA with Tukey’s multiple comparisons. *WT(control)*: *WT(IR)* ****p < 0.0001). **D** Intestinal PMK-1 rescues the abrogated apoptosis phenotype in the *pmk-1(km25)* mutant animals in response to IR. (Two-way ANOVA with Tukey’s multiple comparison test. *WT(IR)*: *pmk-1(IR)* **p = 0.0077, *WT(IR)*: *pmk-1; acEx102(IR)* ns: *p* = 0.5979). **E** Tissue-specific RNAi of *pmk-1* shows that intestine-specific knockdown of *pmk-1* mimics the apoptotic defect of ubiquitous *pmk-1* knockdown, *EV* treatment served as control. (Two-way ANOVA with Tukey’s multiple comparison test. *WT(IR)*: *sun-1p::rde-1;rde-1(IR)* ****p < 0.0001, *WT(IR)*: *nhx-2p::rde-1;rde-1(IR)*, ns: *p* = 0.9999) For **B**, **D**, **E**, *n* = 3 independent experiments, values show mean ± s.e.m. **F** *pmk-1(km25)* mutant animals display reduced levels of apoptosis in response to persistent recombination-mediated DNA damage inflicted by the *syp-2(ok307)* mutation, which can be reverted by intestine-specific expression of PMK-1 under the control of the *vha-6* promoter. *n* = 4 independent experiments, values show mean ± s.e.m. (One-way ANOVA with Tukey’s multiple comparison. *syp-2:syp-2;pmk-1* *p = 0.0365, *syp-2;pmk-1:syp-2;pmk-1;acEx102* ***p = 0.0004). Full statistical results and exact *p* values are shown in the Source data files.

To characterize the role of PMK-1/p38 stress signaling in DNA damage-induced germ cell apoptosis in *C. elegans*, we used the mutant *pmk-1(km25)* and exposed it to 90 Gy of IR. To detect apoptotic corpses, worms were immobilized and germlines were visualized under the Nomarski, differential interference contrast (DIC) microscopy. Apoptotic corpses in the *C. elegans* germline loop can be monitored during the late pachytene stage of meiosis prophase I and can be distinguished from normal germ cells through a distinct morphological change into a disc-shaped format and an increased refractivity^3,29,30^. In line with previous reports, the germ cell death upon IR-induced DNA damage was blunted in *pmk-1(km25)* but not in *jnk-1(gk7)* mutant worms (Fig. 1A, B)^28^. Next, we hypothesized that PMK-1 could regulate germ cell apoptosis through its known downstream transcription factor ATF-7, an ortholog of mammalian ATF2/ATF7^31^. Indeed, *atf-7(qd22*,*qd130)* loss-of-function mutant worms recapitulated the apoptotic defect of *pmk-1(km25)* mutants in response to DNA damage (Fig. 1B). Therefore, we conclude that PMK-1/p38 MAPK signaling is required to induce germ cell apoptosis in response to DNA damage through its downstream transcription factor ATF-7.

Taken into account that PMK-1 expression appears to be restricted to somatic tissues including neurons and the intestine and that intestinal PMK-1 particularly regulates innate immunity against pathogen infection^25,26^, we wondered whether PMK-1-mediated signals from the intestine to the germline might impact germ cell apoptosis in response to DNA damage through a non-cell-autonomous mechanism. In order to test this possibility, we first verified the expression pattern of PMK-1 by using an antibody specifically against an evolutionary conserved PMK/p38 phosphorylation site (p-PMK-1), to detect the active form of PMK-1/p38. Consistent with previous reports, our immunofluorescence result confirmed that the p-PMK-1 is expressed exclusively in somatic cells, particularly the anterior end of the intestine, while this signal is completely abolished in the *pmk-1* mutant (Fig. 1C). Intriguingly, upon IR-induced DNA damage, the level of p-PMK-1 is significantly enhanced (Fig. 1C), and no longer restricted to the anterior end, but instead detectable throughout the nuclei of the intestinal cells (Fig. 1C). Consistent with our immunofluorescence result, we found increased p-PMK-1 levels compared to non-treated WT animals 2 to 3 h post-IR treatment, indicating its activation after IR-induced DNA damage (Supplementary Fig. 1A). In order to investigate the role of intestinal PMK-1, we employed a transgenic strain expressing PMK-1 under the control of the intestine-specific *vha-6* promoter^32^ in the *pmk-1(km25)* mutant background (Supplementary Fig. 1B). In line with our hypothesis, intestine-specific PMK-1 expression in the *pmk-1(km25)* mutant was sufficient to restore germ cells apoptosis upon IR-induced DNA damage (Fig. 1D).

To independently validate the tissue-specific role of PMK-1, we next performed a tissue-specific RNA interference (RNAi) experiment. To this end, we employed *rde-1* mutant strains whose RNAi deficiency is rescued by cell type-specific expression of RDE-1 expressed either exclusively in the germ cells or the intestine. The promoters of *nhx-2* and *sun-1* were previously used to drive tissue-specific expression of RDE-1 in the intestine and germline, respectively^33–35^. RNAi knockdown of *pmk-1* in the intestine, but not in the germline, resulted in a similar reduction of IR-induced germ cell apoptosis as ubiquitous knockdown of *pmk-1* (Fig. 1E). These data indicate that intestinal PMK-1/p38 MAPK signaling is essential to mediate DNA damage-induced germ cell apoptosis through a non-cell-autonomous signaling pathway.

Next, we investigated whether the PMK-1/p38 MAPK stress signaling pathway induces apoptosis not only upon exogenously induced DSBs but also upon endogenous meiosis-specific DSBs. To address this question, we crossed *pmk-1(km25)* to *syp-2(ok307)* mutant worms that fail to properly form the synaptonemal complex during meiosis and lack accurate homologous chromosome alignment thus leading to persistent DSBs specifically in meiotic pachytene cells^13^. We observed that in contrast to *syp-2(ok307)* single mutant worms that were previously shown to exhibit high baseline apoptosis in germ cells, *syp-2(ok307); pmk-1(km25)* double mutant displayed significantly reduced levels of germ cells apoptosis (Fig. 1F). To independently verify that PMK-1 was required for the apoptotic response to endogenous DSBs in germ cells, we used also RNAi against the *syp-2* and *rad-51* genes. Loading of RAD-51 onto DSBs formed during meiosis promotes DNA strand invasion/exchange, which is an essential requirement for efficient homology-directed repair^36^. Similarly to the absence of SYP-2, deficiency in *C. elegans* RAD-51 function contributes to excessive unrepaired DSBs and consequently increased level of apoptosis in germ cells^37^. RNAi knockdown of either *rad-51* or *syp-2* resulted in elevated germ cell apoptosis in wildtype (WT) but not in *pmk-1(km25)* mutant worms (Supplementary Fig. 1C). These data indicate that PMK-1/p38 regulates germ cell apoptosis not only in response to exogenous DSBs but also in response to persistent meiotic DNA damage. Moreover, the requirement for PMK-1 for the induction of apoptosis amid DSBs that specifically occur only in meiotic pachytene cells excludes the possibility that PMK-1/p38 might increase apoptosis as a side effect of IR damage in somatic tissues and not directly due to DSBs in the *C. elegans* germline. Intriguingly, expression of the *acEx102 [vha-6p::pmk-1::gfp]* transgene in the *syp-2(ok307); pmk-1(km25)* double mutant background was sufficient to revert the reduced number of germ cell apoptosis observed in *syp-2(ok307); pmk-1(km25)* worms (Fig. 1F). Therefore, PMK-1/p38 signaling in the intestine is responsible to regulate germ cell apoptosis not only upon IR-mediated DNA damage but also upon meiotic DNA damage occurring specifically in the meiotic pachytene cells.

### PMK-1 regulates apoptosis in parallel or downstream of CEP-1

We next examined at which level PMK-1 signaling impacts the DDR. While the apoptotic DDR in *C. elegans* is strictly limited to meiotic pachytene cells, DNA damage checkpoint signaling, in addition, mediates cell cycle arrest in mitotically dividing cells in the distal germline compartment. Moreover, the DNA damage checkpoint is required for DNA repair; thus, DNA damage checkpoint defects result in failure of mitotic cells to arrest, meiotic pachytene cells to undergo apoptosis, and highly elevated embryonic lethality of embryos formed by the damaged germ cells^3,29,30^. Comparison of F1 embryo survival of the IR-treated WT versus *pmk-1(km25)* and *atf-7(qd22*,*qd130)* parental generation revealed no significant differences (Supplementary Fig. 2A). This observation suggests that PMK-1 does not impact DSB repair. Furthermore, we tested the effect of PMK-1 on cell cycle arrest regulation, which can be scored in the very distal part of the germline by counting the number of proliferating cells in the mitotic zone. As it was established previously, the number of proliferating mitotic germ cells is decreasing gradually in an IR dosage-dependent manner^3,29,30^. The same decline in the number of proliferating mitotic germ cells upon IR as in WT was also observed in the *pmk-1(km25)* as well as *atf-7(qd22*,*qd130)* mutant worms (Supplementary Fig. 2B). Thus, PMK-1 signaling is dispensable for the regulation of cell cycle arrest during the DDR.

We next sought to further characterize the involvement of PMK-1 signaling in the apoptotic DDR. As we have shown, IR irradiation can trigger an increase of p-PMK-1 compared to non-treated WT 2 or 3 h post-treatment (Supplementary Fig. 1A). However, the elevated phosphorylation of PMK-1 upon DNA damage induction was not changed in worms that were deficient for the DNA damage checkpoint protein MRT-2/RAD1 (Supplementary Fig. 1A). In summary, we conclude that the upstream components of the DNA damage checkpoint pathway are not affected by PMK-1/p38 MAPK activity. Instead, PMK-1/p38 is specifically involved in the apoptotic branch of the DDR.

DNA damage-induced apoptosis requires the CEP-1/p53-mediated transcriptional induction of the BH3-only genes *egl-1* and *ced-13*^6,38^. To determine whether PMK-1/p38 triggers germ cell apoptosis by affecting CEP-1/p53 activity, we used quantitative reverse transcription-polymerase chain reaction (qRT-PCR) to test whether the suppressed levels of apoptosis upon DNA damage in *pmk-1(km25)* mutants might be due to compromised transcriptional induction of the CEP-1 target genes, *egl-1* and *ced-13*^8,9^. As demonstrated before, the mRNA expression levels of these two BH3-only genes were highly up-regulated in response to IR in WT worms. While in *cep-1(lg12501)* mutants, the induction of *egl-1* and *ced-13* transcription was completely abrogated^6,38^, those BH3-only domain genes were normally induced upon genotoxic stress in *pmk-1(km25)* mutant worms (Supplementary Fig. 2C). The qRT-PCR data thus imply that even though germ cell apoptosis is abrogated in *pmk-1(km25)* mutants in response to DNA damage, CEP-1 transcriptional activity is not modulated by PMK-1 loss. Therefore, this observation suggests that PMK-1/p38 could genetically function in parallel to or downstream of CEP-1/p53 to induce germ cell apoptosis upon IR-mediated DNA damage.

We next addressed the possibility of PMK-1/p38 to have an impact downstream of CEP-1/p53, on the core apoptotic machinery level. We employed a *ced-9(n1653)* loss-of-function mutation that abrogates the activity of the anti-apoptotic Bcl-2 homolog CED-9 and generated a double mutant with *pmk-1(km25)*. The lack of CED-9/Bcl-2 leads to elevated levels of physiological as well as DNA damage-induced apoptosis in *C. elegans*^39,40^. Interestingly, *ced-9(n1653)* loss-of-function was capable of reverting the abrogated apoptosis phenotype of *pmk-1(km25)* with and without IR treatment to an extent similar to *ced-9(n1653)* single mutants (Supplementary Fig. 2D). Therefore, elevated levels of apoptosis in *ced-9(n1653)*; *pmk-1(km25)* double mutants demonstrate that the PMK-1/p38 MAPK signaling pathway functions upstream of core apoptotic machinery to regulate DNA damage-induced germ cell apoptosis, possibly by blocking CED-9/Bcl-2 activity.

### PMK-1 target SYSM-1 (T24B8.5) mediates germ cell apoptosis

**Fig. 2:**
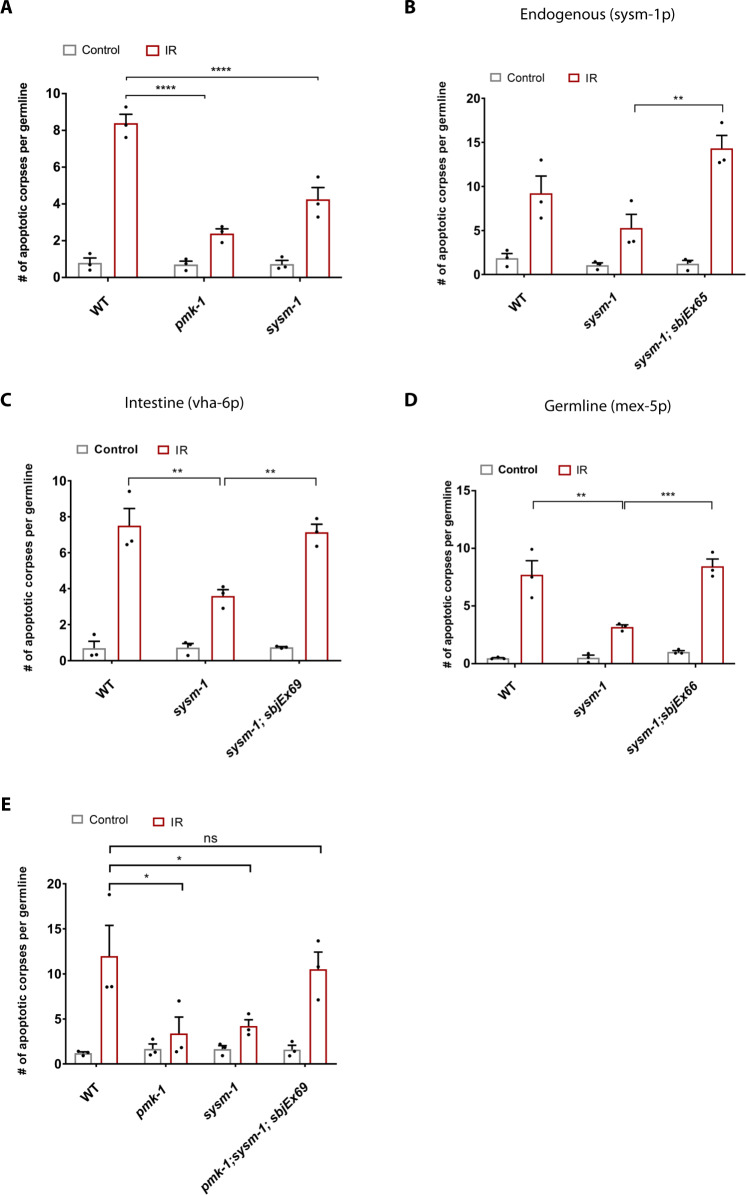
PMK-1/p38 target SYSM-1 regulates germ cell apoptosis non-cell-autonomously. **A** *sysm-1(ok3236)* mutants are defective in inducing apoptosis upon IR treatment similarly to *pmk-1(km25)* mutants. **B** Endogenous-, **C** intestine-, and **D** germline-specific expression of SYSM-1 rescues the apoptosis defect in *sysm-1(ok3236)* mutant worms in response to IR. **E** Intestine-specific expression of SYSM-1 suppresses the apoptotic defect of *pmk-1* mutants. The graphs **A**–**E** display *n* = 3 independent experiments and each dot indicates one independent experiment that includes 8–20 germlines per condition scored 6 h post-IR (90 Gy). Error bars show mean ± s.e.m. (Two-way ANOVA with Tukey’s multiple comparison test. **A** *WT(IR)*: *pmk-1(IR)* ****p < 0.0001, *WT(IR)*:*sysm-1(IR)* ****p < 0.0001, **B** *sysm-1(IR):sysm-1;sbjEx65(IR)* **p = 0.0022, **C** *WT(IR)*:*sysm-1(IR)* **p = 0.0011, *sysm-1(IR):sysm-1;sbjEx69(IR)* **p = 0.0026, **D** *WT(IR)*:*sysm-1(IR)* **p = 0.0013, *sysm-1(IR):sysm-1;sbjEx66(IR)* ***p = 0.0003, **E** *WT(IR)*: *pmk-1(IR)* *p = 0.0231, *WT(IR)*:*sysm-1(IR)* *p = 0.0465, *WT(IR)*:*pmk-1*;*sysm-1;sbjEx69(IR)* ns: *p* = 0.9972). Full statistical results and exact *p* values are shown in the Source data files.

We next wished to determine how intestinal PMK-1/p38 MAPK regulates germ cell apoptosis non-cell-autonomously. It was reported that the *T24B8.5* gene, which contains an N-terminal signal sequence for extracellular secretion as well as a ShK-like toxin domain is robustly up-regulated in intestines of *C. elegans* upon pathogenic bacteria infection in a PMK-1- and ATF-7-dependent manner^31^. Based on the functional characterization below, we propose to name the *T24B8.5* gene *systemic stress signaling mediator (sysm-1)*, which we use from here onwards (a request to wormbase to rename T24B8.5 to *sysm-1* has been approved). We previously showed that germlines exhibit activation of the MPK-1 pathway, which in turn induces *sysm-1* mRNA expression upon IR treatment^22^. *sysm-1* mRNA was indeed induced in response to IR in WT but not *pmk-1* mutant animals (Supplementary Fig. 3A). Consistent with SYSM-1 being a downstream effector of PMK-1 signaling, we found that *sysm-1(ok3236)* mutants showed strongly reduced germ cell apoptosis upon IR treatment (Fig. 2A). To further test whether the putative secreted peptide encoded by the *sysm-1* gene might mediate intestinal regulation of germ cell apoptosis upon genotoxic stress, we constructed transgenic strains expressing *sysm-1* under its endogenous as well as cell type-specific promoters. Given that the SYSM-1::GFP signal was not detectable in transgenic lines possibly due to cleavage of GFP (likely due to processing in the secretory pathway), we performed in situ hybridization on dissected intestines and germlines to confirm the tissue-specific expression of SYSM-1 in the respective tissues (Supplementary Fig. 3B). Re-expression of the SYSM-1 protein fused to a C-terminal GFP under the control of its endogenous promoter not only reverted the apoptosis defect of *sysm-1(ok3236)* mutants but exacerbated the apoptotic response to IR (Fig. 2B). Expression of SYSM-1::GFP under the control of an intestine-specific promoter *vha-6* restored the decreased apoptosis level of SYSM-1 deficiency (Fig. 2C). In addition, germline-specific SYSM-1::GFP, which was constitutively expressed under the *mex-5* promoter^41,42^ was also sufficient to restore IR-induced germ cell apoptosis in the *sysm-1(ok3236)* mutant background (Fig. 2D). These results indicate that (i) SYSM-1 is required, (ii) intestinal SYSM-1 is sufficient, and (iii) constitutive germline expression of SYSM-1 is sufficient for DNA damage-induced germ cell apoptosis.

To verify whether SYSM-1 mediates the apoptotic signaling as downstream effector of PMK-1 in response to DNA damage, we tested whether expression of SYSM-1 in the intestine, using the *vha-6* promoter, could rescue the apoptotic defect of the *pmk-1* mutant. Indeed, constitutive re-expression of SYSM-1 in the intestine was sufficient to restore IR-induced apoptosis in *pmk-1* mutants to WT levels (Fig. 2E). Together, these observations indicate that SYSM-1 functions downstream of PMK-1/p38 MAPK signaling pathway as a putative intestinal secreted immune peptide in response to IR-induced DNA damage to promote apoptosis in germ cells in a non-cell-autonomous fashion.

### Secreted intestinal SYSM-1 acts on germline apoptosis

**Fig. 3:**
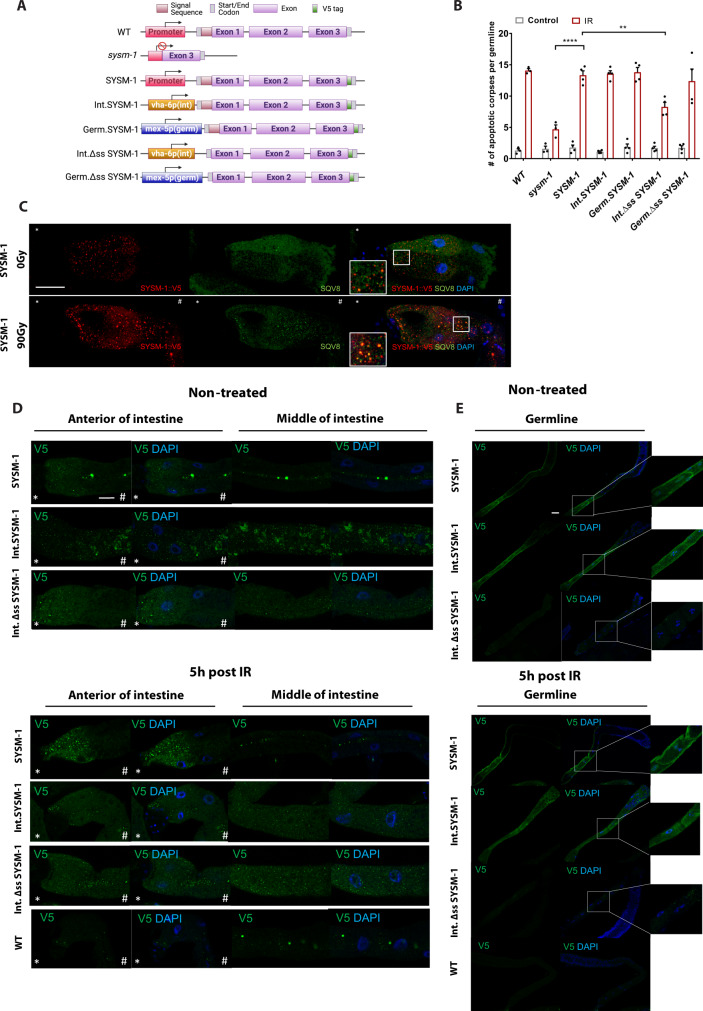
SYSM-1 is secreted from the intestine to regulate apoptosis in the germline upon IR. **A** Schematic representation of *sysm-1* gene in WT, *sysm-1(ok3236)* mutant and the CRISPR/Cas9-edited strains with swapping of the endogenous promoter for intestine- or germline-specific promoters and with WT or deleted signal sequence (ss). **B** Quantification of apoptotic corpses per germline in WT, *sysm-1* and CRISPR/Cas9 strains shown in **A**. The graph summarizes *n* = 4 independent experiments, and each dot indicates one independent experiment. Each experiment includes 10–20 germlines per condition scored 6 h post-IR (90 Gy). Error bar shows mean ± s.e.m. (*p < 0.05, **p < 0.01, ***p < 0.001, ****p < 0.0001, Two-way ANOVA with Tukey’s multiple comparison test. *sysm-1(IR):SYSM-1(IR)* ****p < 0.0001, *SYSM-1(IR): Int.Δss SYSM-1* **p = 0.001). Full statistical results and exact *p* values are shown in the Source data files. **C** Representative images from immunofluorescence co-staining of SYSM-1::V5 (red) and the Golgi marker SQV8 (green). Merged images with DAPI are shown at the right side, and co-localized foci in the frame are zoomed in and shown in the corners. Scale bar = 20 μm. This experiment was repeated two times with similar results. **D** Representative images of SYSM-1::V5 staining in dissected intestines of the indicated strains with or without IR treatment are shown. *Indicates anterior side; ^#^indicates posterior side. Merged images with DAPI are placed at the right side. Scale bar = 20 μm. **E** Representative images of SYSM-1::V5 staining in dissected germlines of the indicated strains with or without IR treatment are shown. Merged images with DAPI are shown at the right side. Oocyte regions in the frame are zoomed in and shown in the corners. Scale bar = 20 μm. WT strain was used as control, only background signal was detected. The experiments in **D**, **E** were repeated three times with similar observations.

To further investigate whether intestinal SYSM-1 mediates the IR-induced apoptosis in the germline, we aimed to visualize the distribution pattern of SYSM-1 upon IR treatment directly. Given that a SYSM-1::GFP fusion was unattainable, we generated a series of CRISPR/Cas9 knock-in strain targeting the endogenous locus. Initially, we constructed a strain carrying a V5 tag at the C-terminal of the endogenous SYSM-1 protein (Fig. 3A). This SYSM-1::V5 strain (SYSM-1) shows the comparable level of apoptosis as WT animals post-IR treatment indicating that the fusion protein is functional (Fig. 3B). Using a V5 tag-specific antibody to detect the distribution pattern of SYSM-1, we observed a punctate pattern of SYSM-1 in the anterior intestinal cells, with strongly elevated levels upon IR treatment (Fig. 3C, D and quantifications in Supplementary Fig. 4D).

As SYSM-1 is a putative secreted peptide, we were wondering if this punctate signal of SYSM-1 co-localizes with the Golgi apparatus. Indeed, by co-staining with Golgi apparatus marker, SQV8, we observed a strong co-localization between SYSM-1 and SQV8, indicating that SYSM-1 is processed and secreted via Golgi apparatus in the anterior part of the intestine (Fig. 3C). In the germline, we also detected a clear, albeit weak, signal of the SYSM-1 peptide (Fig. 3E and Supplementary Fig. 4D). However, the punctate signal of SYSM-1 is not co-localized with SQV8 in germline cells (Supplementary Fig. 4A). Taken together, these results indicate that SYSM-1 is secreted from the intestine into the germline.

As the intestine-specific expression of SYSM-1 rescued the apoptosis defect of *sysm-1* mutant, we next queried whether the intestine-expressed SYSM-1 regulates germline apoptosis via secretion. To answer this question, we generated two CRISPR/Cas9 knock-in strains replacing the endogenous promoter with the intestinal-specific *vha-6* promoter and either retained or deleted the signal sequence (ss) of SYSM-1 (Fig. 3A). We first performed immunofluorescence to verify the distribution pattern of intestine-expressed SYSM-1 and ∆ss SYSM-1. We found that the *vha-6* promoter-driven SYSM-1 is distributed throughout the intestine and was also detectable in the germline (Fig. 3D, E). In stark contrast, intestine-expressed ∆ss SYSM-1 was only detectable in intestinal cells, but completely abolished in germline (Fig. 3D, E and Supplementary Fig. 4D). These results establish that SYSM-1 that is generated in the intestine is secreted into the germline through recognition of the signal sequence.

To address whether secretion of SYSM-1 is required for mediating germ cell apoptosis we quantified cell death in the SYSM-1 strains. While SYSM-1::V5 was similarly proficient as intestinal and germline expressed SYSM-1, intestinal expression of SYSM-1 lacking the secretory signal sequence significantly reduced germ cell apoptosis (Fig. 3B). Therefore, SYSM-1 is secreted from the intestine into the germline where is required to facilitate DNA damage-induced apoptosis.

To test whether the presence of SYSM-1 in the germline was sufficient to trigger apoptosis independently of secretion, we generated two CRISPR/Cas9 strains carrying SYSM-1 or ∆ss SYSM-1 under the control of germline-specific promoter, *mex-5p* (Fig. 3A). Unlike the SYSM-1 expressed under the control of the endogenous or intestine-specific promoters, germline promoter-driven SYSM-1 was undetectable in intestinal cells but strongly expressed in germline (Supplementary Fig. 4B, C). Germline specifically expressed SYSM-1 and ∆ss SYSM-1 were both able to induce apoptosis upon IR treatment similarly to WT (Fig. 3B). These results establish that SYSM-1 acts in the germline to facilitate DNA damage-induced apoptosis and that the signal sequence is dispensable for the apoptotic function when SYSM-1 is expressed in the germline.

The ERK MAPK MPK-1 was previously shown to regulate DNA damage-induced apoptosis in meiotic pachytene cells^43^ and exert systemic effects by triggering germline DNA damage-induced systemic stress resistance (GDISR)^22^. We, therefore, wondered whether MPK-1/ERK signaling might interact with intestinal PMK-1/p38 signaling to regulate germ cell apoptosis. While *mpk-1* mutants displayed elevated baseline p-PMK-1 levels in the intestine, they failed to induce intestinal p-PMK-1 further upon IR treatment (Supplementary Fig. 5A, B). We next probed for the effect of hyperactivation of MPK-1/ERK. We previously showed that ALG-2 represses MPK-1/ERK activation and that *alg-2* mutants show strongly exacerbated MPK-1/ERK activation in meiotic pachytene cells upon DNA damage^44^. However, *alg-2* mutants displayed a normal IR-dependent PMK-1/p38 activation in the intestine, which was suppressed in *mpk-1;alg-2* double mutants consistent with the pro-apoptotic role of MPK-1/ERK downstream of ALG-2. These data indicate a feedback loop between germline MPK-1/ERK and intestinal PMK-1/p38 where in the absence of genotoxic stress in germ cells, MPK-1/ERK limits intestinal PMK-1/p38 activation. Hyperactivation of MPK-1/ERK in *alg-2* mutants, however, was able to suppress the apoptotic defect of *sysm-1* indicating that the MPK-1/ERK-mediated activation of the apoptosome in germ cells operates downstream of PMK-1/p38-SYSM-1 signaling (Supplementary Fig. 5C). These data suggest that germline MPK-1/ERK functions downstream of the intestinal apoptotic input and reversely curbs intestinal PMK-1/p38 activation in the absence of genotoxic stress.

### PMK-1 maintains heritable genomic diploidy upon heat stress

**Fig. 4:**
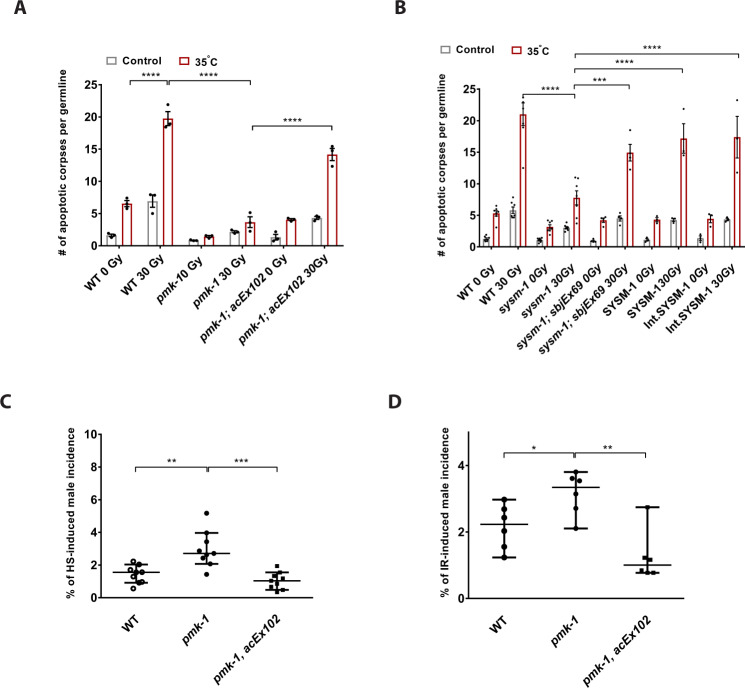
Intestinal PMK-1/p38 induces germ cell apoptosis and maintains heritable euploidy upon heat stress and IR treatment. **A** Transient heat stress further promotes IR-induced germ cell apoptosis in an intestinal PMK-1-dependent manner. The graph summarizes *n* = 3 independent experiments, and each dot indicates one independent experiment. Each experiment includes 8–15 germlines per condition scored 6 h post-IR (Young adult worms were heat shock treated at 35 °C for 10 min and subsequently exposed to 30 Gy of IR). Error bars show mean ± s.e.m. (Two-way ANOVA with Tukey’s multiple comparison test. *WT(0* *Gy)35* *°C:WT(30* *Gy)35* *°C* ****p < 0.0001, *WT(30* *Gy)35* *°C:pmk-1(30* *Gy)35* *°C* ****p < 0.0001, *pmk-1(30* *Gy)35* *°C:pmk-1; acEx102(30* *Gy)35* *°C* ****p < 0.0001). **B** Transient heat stress further promotes IR-induced germ cell apoptosis in an intestinal SYSM-1-dependent manner. The graph summarizes *n* = 7 independent experiments for the WT and *sysm-1*, n = 4 independent experiments for *sysm-1; sbjEx69*, n = 3 independent experiments for SYSM-1 and Int. SYSM-1, and each dot indicates one independent experiment. Young adult worms were heat shock treated at 35 °C for 10 min and subsequently exposed to 30 Gy of IR. Error bars show mean ± s.e.m. (Two-way ANOVA with Tukey’s multiple comparison test, *WT(30* *Gy)35* *°C:sysm-1(30* *Gy)35* *°C* ****p < 0.0001, *sysm-1(30* *Gy)35* *°C:sysm-1;sbjEx69(30* *Gy)35* *°C* ***p = 0.0001, *sysm-1(30* *Gy)35* *°C:SYSM-1(30* *Gy)35* *°C* ****p < 0.0001, *sysm-1(30* *Gy)35* *°C:Int.SYSM-1(30* *Gy)35* °C ****p < 0.0001). **C** Transient heat stress at 30 °C elevates the percentage of male incidence in the F1 generation of *pmk-1(km25)* mutant animals, which can be rescued by intestinal-specific PMK-1 expression in *pmk-1(km25); acEx102* transgenic lines (L4 worms heat shock treated at 30 °C for 6 h and then shifted to 20 °C). The graph summarizes *n* = 9 independent experiments each performed in biological triplicate per genotype (Sample size: *N* = 10 adult worms used for 30 h of egg-laying experiment). (Mann–Whitney non-parametric test. *pmk-1:WT* **p = 0.0012, *pmk-1;acEx102:pmk-1* ***p = 0.0002). **D** 90 Gy of IR treatment results in an increased percentage of male incidence in the F1 generation of *pmk-1(km25)* mutant animals, which can be restored by intestine-specific PMK-1 expression in *pmk-1*-deficient worms (L4 worms were exposed to 90 Gy of IR). The graph summarizes *n* = 6 independent experiments each performed in biological triplicate per genotype (Sample size: N = 10 adult worms used for 30 h of egg-laying experiment). (Mann–Whitney non-parametric test. *pmk-1:WT* *p = 0.026, *pmk-1;acEx102:pmk-1* **p = 0.0087). Medians with 95% CI are shown in **C**, **D**. Full statistical results and exact *p* values are shown in the Source data files.

We next wondered whether suppressed DNA damage-induced apoptosis due to loss of PMK-1/p38 could affect the genomic integrity of the following generation. Therefore, we wished to clarify whether PMK-1/p38 MAPK signaling and its role in DNA damage-induced germ cell apoptosis might also be responsible for accurate meiotic chromosome segregation in response to physiological stressors. PMK-1 stress signaling has been established to regulate responses not only to pathogenic infection and oxidative stress but also upon heat stress^24^. Therefore, we evaluated first whether PMK-1 might regulate germ cell apoptosis in response to somatic stress conditions such as heat stress. We therefore pre-conditioned WT and *pmk-1(km25)* mutant worms with transient heat stress at 35 °C, before treating them with IR. This short and acute heat stress was not sufficient to increase X chromosome nondisjunction as male incidence was not seen in the F1 generation of WT and *pmk-1(km25)* mutant worms. Furthermore, the number of viable F1 progeny was not altered, while WT and *pmk-1(km25)* mutant parental worms were exposed to the transient heat stress, suggesting no potential meiotic failure and aneuploidy in the next generation (Supplementary Table 1). Instead, this transient heat stress led to increased germ cell apoptosis that was further exacerbated upon IR treatment. As under heat stress, the worms have higher apoptosis levels, we limited the IR here to 30 Gy. Therefore, we propose that the heat stress response synergizes with the DDR in the induction of germ cells apoptosis (Fig. 4A). Intriguingly, the heat stress-fueled apoptotic response was blunted in *pmk-1(km25)* mutant animals. Specific transgenic re-expression of PMK-1 in the intestine of *pmk-1(km25)* mutant worms significantly restored the heat stress-induced exacerbation of germ cell apoptosis (Fig. 4A).

In addition to PMK-1, we next examined the role of SYSM-1 in response to heat stress. Similar to *pmk-1(km25)*, *sysm-1(ok3236)* also shows a significant reduction of apoptosis in response to heat stress combined IR treatment, and it can be rescued by the transgenic re-expression of SYSM-1 in the intestine of *sysm-1(ok3236)* (Fig. 4B). Moreover, intestine-specific expression of SYSM-1 is sufficient to induce heat stress-fueled apoptosis response (Fig. 4B). Taken together, we demonstrate that the intestinal PMK-1/p38 and SYSM-1 impact DNA damage-induced germ cell apoptosis upon heat stress. These results suggest that PMK-1 signaling relays somatic stress responses to impact the regulation of germ cell apoptosis through a non-cell-autonomous stress surveillance mechanism.

Next, we assessed whether the intestinal PMK-1/p38 MAPK-mediated germ cell apoptosis in response to heat stress could impact euploidy of the next generation. Chromosome nondisjunction in *C. elegans* can result in missegregation of the X chromosome, which determines nematode sex as either hermaphrodites (XX) or less frequently, males (XO)^45^. Exposure of *C. elegans* hermaphrodites to 30 °C for a time-period of 6 h results in the high incidence of males known as HIM phenotype in the population^46^. Intriguingly, we found that heat stress exposure of *pmk-1(km25)* mutant worms at 30 °C led to a robust increase in male incidence indicating inheritance of missegregated X chromosomes. The intestinal PMK-1 re-expression was more than sufficient to restore the male incidence to a level comparable to WT worms (Fig. 4C). In addition to heat stress, we observed that *pmk-1(km25)* mutant worms had an elevated level of male incidence in response to IR treatment which was rescued by re-expression of PMK-1 in the intestine of PMK-1-deficient worms (Fig. 4D).

In contrast to *pmk-1* mutants, *sysm-1(ok3236)* mutants showed a strongly diminished brood size. The reduced fecundity of *sysm-1* mutants was rescued by mating with WT males thus indicating a severe defect in spermatogenesis (Supplementary Fig. 6), which is further deteriorated upon heat stress or IR treatment. Only the first ~ 10 oocytes could be self-fertilized thus precluding the analysis of aneuploidy in the offspring following heat stress or DNA damage.

In conclusion, intestinal PMK-1/p38 MAPK signaling is required for eliminating genomically compromised germ cells to ensure heritable genomic diploidy amid environmental or genotoxic stress.

## Discussion

The DNA damage checkpoint during meiosis is essential to ensure the inheritance of a stable and euploid genome. In mammals, p38 MAPK signaling regulates stress responses ranging from inflammation to DNA damage checkpoint signaling^47^. In this study, we demonstrate that intestinal PMK-1/p38 regulates germ cell apoptosis in response to exogenous and endogenous DSBs during meiosis. Intriguingly, we found that PMK-1/p38 exacerbates DNA damage-induced germ cell apoptosis in response to heat stress suggesting that PMK-1/p38 in the intestine relays somatic stress surveillance to the regulation of germ cell apoptosis and thus to the control of the integrity of heritable genomes. PMK-1/p38 in *C. elegans* was established as stress and innate immune responsive MAPK pathway. PMK-1/p38 in intestinal cells is required for the worm’s survival of *Yersinia pestis* infections^27^. In addition, the upstream PMK-1 MAPKK, SEK-1 was shown to function in the intestine and in chemosensory neurons to confer resistance to *P. aeruginosa*^26^. The second downstream SEK-1 MAPKK target, PMK-2 was found to modulate the behavioral response to pathogens together with PMK-1 in neurons, whereas intestinal PMK-1 alone was essential to regulate innate immune responses to pathogens^25^. Germ cell apoptosis in *C. elegans* is indeed controlled non-cell-autonomously even though the mechanisms and particularly the consequences of the somatic influences have remained poorly understood. For example, neuronal HIF-1 and IRE-1 regulate CEP-1/p53, while intestinal KRI-1 functions in parallel to CEP-1/p53, but upstream of CED-9/Bcl-2 to regulate germ cell apoptosis in response to IR-induced DNA damage^48–50^. We show here that PMK-1/p38 affects germ cell apoptosis upstream of the CED-9/Bcl-2 components of the core apoptotic machinery.

We have identified the PMK-1 target gene *sysm-1* that encodes a secreted peptide and mediates the non-cell-autonomous control of germ cell apoptosis, via secretion from the intestine into the germline. We determined that endogenous p-PMK-1 and SYSM-1 are both mainly expressed at the anterior end of the intestine (Figs. 1C and 3C, D). The anterior end of the intestine is anatomically located in close proximity to the bend region of germline, which is the only section of the germline where late pachytene cells undergo apoptosis. It is thus conceivable that the anterior intestinal cells secrete SYSM-1 directly into the germline to promote germ cell apoptosis in close proximity. Given that both PMK-1/p38 and MPK-1/ERK MAPKs are required for the induction of SYSM-1 in response to DNA damage it is likely that both MAPK signaling pathways interact across the germline and the soma (Supplementary Fig. 3A)^22^. We established previously that MPK-1/ERK exerts multiple roles in the DDR including triggering GDISR^22^ and facilitating apoptosis upon initial activation of the apoptosome in germ cells^44^. Our data suggest that in the absence of genotoxic stress, MPK-1/ERK curbs the intestinal activation of PMK-1/p38, and only upon DNA damage the two MAPK pathways are triggered and PMK-1-induced SYSM-1 mediates the MPK-1/ERK to fully activate the apoptosome in germ cells.

It is conceivable that SYSM-1 directly or indirectly impinges on CED-9/Bcl-2 activity potentially at the surface of mitochondria to block its anti-apoptotic effect in response to DNA damage. In mammals, cytokines have been reported to impinge on apoptosis by affecting the expression levels of Bcl-2 and Bcl-xL in myeloid leukemia cells^51^. Cytokine-mediated regulation of Bcl-2 levels and thus the balancing of apoptosis is of fundamental importance also in the maternal-fetal interaction during pregnancy. In humans, the pro-inflammatory cytokine tumor necrosis factor-α (TNFα) has been shown to modulate the Bcl-2 and Bax ratios thus affecting apoptosis in the endometrium and placenta. The perturbation of apoptosis by an increase of TNFα in the placenta has been linked to early pregnancy failure and premature labor in mouse, rat, and human. Importantly, various abnormal karyotypes can be observed in approximately 60% of miscarriage cases^52^. It will be important to investigate whether dysregulation of apoptosis by a disturbance of the cytokine balance might be responsible for these abnormal karyotypes.

To prevent infertility, miscarriage, and birth pathologies, the maintenance of proper numerical chromosome composition across generations is of critical importance. In *C. elegans*, apart from the naturally occurring missegregation of X chromosomes in healthy XO males, trisomy of the X chromosome has been observed in the offspring of *him* mutants with various meiotic failures. Trisomy of the X chromosome results in viable worms, although with a dumpy phenotype and lower brood size and reduced fertility. Autosomal chromosome aneuploidy was first reported in the F1 generation of *him-6* mutants due to non-disjunction of chromosome IV with no obvious defect on overall physiology but reduced fertility^45,53^. In *Drosophila melanogaster*, the JNK MAPK signaling pathway inhibits the increase of aneuploidy through modulation of the pro-apoptotic gene *hid*, albeit only in adult cells upon IR-induced DNA damage in a p53-independent manner^54^.

We propose that the HIM phenotype and aneuploidy of the X chromosome is dependent on intestinal PMK-1/p38 signaling upon heat or genotoxic stress. To our knowledge, there have never been reports of somatic stress signaling pathways affecting the heritable chromosome copy numbers in an animal species. It has been established that excessive heat exposure has negative effects on human male reproductive tissues and the spermatogenesis process and could cause infertility^55^. Moreover, the infertility of the Akita diabetic mice can be restored by exogenous insulin treatment. This systemic hormonal rescue of male infertility, despite incapability of passing through the blood–testis barrier, functions via the hypothalamic–pituitary–gonadal (HPG) axis to restore testosterone levels in the testis^56^. Recent human and animal studies have illustrated the profound negative effects of various types of prenatal stresses on gestation and physiological development of the fetus and the health span of the resulting offspring. Direct effects of stress on pregnancy can be observed via alteration in endocrine activity as well as inflammatory responses^57^. It was suggested that stress can modulate the hypothalamo-pituitary-adrenal (HPA) axis to elevate the production of corticotropin-releasing hormone, which in turn contributes to the induction of inflammatory cytokines during gestation^58^. Maternal stress can also increase the glucocorticoid levels in utero, which can evoke stress responses in the fetus with negative effects on the development and physiology of the offspring^59^. Our study indicates an important role of somatic stress surveillance via the intestinal PMK-1/p38 signaling pathway in the regulation of meiotic chromosomal quality control and thus the inheritance of stable genomes. The Weismann barrier concept pertained that the germline is protected from somatic influences and thus genetic inheritance is only controlled autonomously by germ cells^60^. Our data challenge the Weismann barrier as we propose that somatic stress surveillance regulates the inheritance of chromosomes via the DDR control of the apoptotic elimination of aneuploid gametes.

## Methods

### *C. elegans* strains

All strains were maintained based on standard conditions at 20 °C^61,62^. Strains used were N2 (Bristol; wildtype), KU25 *pmk-1(km25)*, VC8 *jnk-1(gk7)*, ZD318 *atf-7(qd22*,*qd130)*, AV276+*/nT1[qIs51](IV); syp-2(ok307)/nT1[qIs51](V)*, BJS717 *syp-2(ok307) V/nT1[qIs51](IV); pmk-1(km25)IV*, AY102 *pmk-1(km25) IV; acEx102 [vha-6p::pmk-1::GFP*+*rol-6(su1006)]*, CB5348 *mrt-2(e2663)*, XY1054 *cep-1(lg12501)*, PP935 *ced-9(n1653)*, BJS373 *pmk-1(km25); ced-9(n1653)*, VC2477 *sysm-1 (ok3236)*, SD939 *mpk-1(ga111) unc-79(e1068);* WM53 *alg-2 (ok304);* BJS710 *sysm-1(ok3236); sbjEX65 [sysm-1p::SYSM-1::GFP*+*myo-2::tdTomato]*, BJS751 *sysm-1(ok3236); sbjEx66 [mex-5p::SYSM-1::GFP*+*myo2::dtTomato]*, BJS818 *sysm-1 (ok3236); sbjEx69 [vha-6p::SYSM-1::GFP*+*myo-2::tdTomato]*, BJS844 *syp-2(ok307) V/nT1[qIs51](IV); pmk-1(km25)IV; acEx102 [vha-6p::pmk-1::GFP*+*rol-6(su1006)]*; BJS984 *pmk-1(km25)*; *sysm-1 (ok3236); sbjEx69 [vha-6p::SYSM-1::GFP*+*myo-2::tdTomato];* BJS985 *sysm-1 (ok3236); alg-2 (ok304);* BJS608 alg-2(ok304);mpk-1(ga111) unc-79(e1068). 5 strains were generated by SunyBiotech (China) and verified with genotyping PCR, they are: PHX3100 *sysm-1(syb3100 [vha-6p::sysm-1::V5]); PHX3078 sysm-1(syb3078[mex-5p::sysm-1::V5]); PHX3073 sysm-1(syb3073[vha-6p::sysm-1(∆ss)::V5]); PHX 3060 sysm-1(syb3060[mex-5p::sysm-1(∆ss)::V5]); PHX2848 sysm-1(syb2848[sysm-1::V5])*.

### Quantification of meiotic germ cell apoptotic corpses

To quantify the number of apoptotic corpses in the meiotic late pachytene germ cells in response to DNA damage, the synchronized L4 worms were separated from the rest of the population and 20 h later at the young adult stage were either remained untreated or exposed to 90 Gy of IR inflicted by a cesium 137 irradiation source (Biobeam GM 8000, Eckert & Ziegler, Gamma-Service Medical GmbH)^3^. 6 h post-IR exposure, worms were immobilized using 5 mM levamisole (AppliChem #A431005) and mounted on a 2% agarose pad on a microscope slide. The number of apoptotic corpses was scored via Nomarski DIC microscopy on a Zeiss Axio Imager M1/2 based on the refractive morphological changes occurring in apoptotic germ cells within the gonad loop^29,63^. For quantification of heat stress-induced apoptosis, young adult worms on NGM plates were exposed for 10 min to heat shock at 35 °C in a pre-heated water bath and were treated with 30 Gy of IR. The number of apoptotic corpses was subsequently counted 5 h post-IR treatment via the same procedure as mentioned above.

### Assessing mitotic germ cell cycle arrest

To assess the mitotic germ cell cycle arrest response to DNA damage, synchronized L4 larva were exposed to IR doses of 0, 30, 60, and 90 Gy. Worms were immobilized using 5 mM levamisole and mounted on 2% agarose pads for Nomarski DIC microscopy 16 h post-IR treatment. A defined region of the distal germline can be scored for either mitotic proliferative cells under normal conditions or the presence of arrested enlarged germ cell nuclei in response to IR damage. This defined field corresponds to an area of 3.125 μm × 6.25 μm in the most distal region of the germline. For precise counting of mitotic germ cells within a specific scale, a Netmicrometer was applied to the microscope ocular (*d* = 26 mm, 12.5 × 12.5/5;10, Zeiss) and the germ cell nuclei in all focal planes were counted using the Zeiss Axio Imager M1/2^3,10,29^.

### Measurement of embryonic lethality

Synchronized L4 animals were separated from the rest of the worm population by transferring them to fresh OP50-seeded plates via picking and were subsequently IR-treated with 0, 30, and 60 Gy. Sixteen hours post-IR exposure, 5 worms from each condition were transferred to three separate OP50-seeded plates to serve as three biological replicates and were allowed to lay eggs for 2 h. The mother worms were then removed from the plates and the number of eggs was counted. The number of surviving offspring was quantified 48 h after the egg-laying process to examine the embryonic hatching rate and DNA damage repair efficiency^3,29^.

### RNAi treatment

For depletion of *pmk-1*, *rad-51,* and *syp-2* gene activities, worms were fed on HT115 *E. coli* bacteria harboring the RNAi plasmid (L4440) which expressed dsRNA against the desired genes from the Ahringer library. The RNAi plasmids were extracted (Nucleospin-Plasmid kit, MACHEREY-NAGEL), sequenced, and then transformed into fresh HT115 *E. coli* competent cells. A single colony was then selected and grown in LB medium supplemented with 50 μg/ml ampicillin at 37 °C to yield an overnight culture. The next day, the HT115 overnight culture was used to inoculate a fresh culture supplemented with ampicillin that was subsequently incubated at 37 °C until reaching an OD of 0.6. The HT115 bacteria containing the respective RNAi plasmids were then induced with 1 mM IPTG to trigger the production of dsRNA for 3 h at 37 °C. The bacteria were then pelleted with high-speed centrifugation and 1/3 of the supernatant was removed. The bacterial pellet was re-suspended in the residual LB supernatant and seeded on NGM plates containing 1 mM IPTG and was incubated overnight at room temperature^64^. Synchronized L1 worms of the respective genotypes were transferred to these RNAi plates and were allowed to grow at 20 °C. Counting of apoptotic corpses was performed 20 h post L4 stage using Nomarski DIC microscopy.

### Heat-stress and IR-induced male incidence

To evaluate X chromosome missegregation and to measure male incidence rate in *C. elegans*, synchronized worms at L4 larval stage were transferred to fresh OP50-seeded plates and exposed to 90 Gy of IR or heat stress in a 30 °C incubator for 6 h^46^. 16 h post-heat stress, 10 worms per each genotype were transferred to three separate OP50-seeded plates to serve as three biological replicates and were allowed to lay eggs for 5 consecutive hours. Worms were subsequently transferred to fresh OP50-seeded plates and were allowed to continue egg laying for another 25 h before removing the mothers from the plates (The analysis was limited to the first 30 h of egg laying since heat-stressed mothers proceeded to lay unfertilized oocytes after this time window). The number of surviving offspring and the number of males in the population was counted 72 h post-egg laying. The percentage of male incidence was quantified and compared between each genotype using the data from 9 and 6 independent experiments in Fig. 3B and C respectively.

### Plasmid constructs and generation of transgenic lines

For the generation of tissue specific and ubiquitous SYSM-1 translational reporter lines, *pPD95.75*, a GFP containing backbone vector, was used (Fire Lab *C. elegans* Vector Kit, Addgene #1494). *sysm-1*genomic DNA excluding its stop codon was PCR amplified using Phusion High-Fidelity DNA-Polymerase (NEW ENGLAND BioLabs #M0530S) and wildtype N2 genomic DNA as a template. Promoter sequences were obtained from the *C. elegans* Promoterome Database^65^. To achieve ubiquitous expression of SYSM-1 protein with a C-terminal GFP-tag, the plasmid *pBS279* was constructed by inserting the *sysm-1* genomic DNA into the *pPD95.75* backbone vector. The *sysm-1* promoter was inserted upstream of *sysm-1::gfp*. To confer germline-specific expression, the *pBS353* vector was generated by inserting a *mex-5* promoter into the *sysm-1::gfp* containing backbone vector. Furthermore, the *sysm-1* intestinal-specific rescue plasmid *pBS360* was constructed by inserting the *vha-6* promoter upstream of *sysm-1::gfp*. *sysm-1(ok3236)* worms were then injected in the germlines with 2.5 ng/µl of *pBS279* or *pBS353* or *pBS360* and 25 ng/µl of pBS174, myo-2::tdTomato as a microinjection marker, to generate extrachromosomal array of *sbjEX65 [SYSM-1P::SYSM-1::GFP* + *myo-2::tdTomato]*, *sbjEx66 [mex-5P::SYSM-1::GFP* + *myo2::dtTomato]*, and *sbjEx69 [vha-6P::SYSM-1::GFP* + *myo-2::tdTomato]*, respectively. The following forward and reverse primers were used: *sysm-1* promoter: 5´aaaaccggttttggtgatacataaatgataa3´, 5´ttttctagaaactggacattttccctc3´; *vha-6* promoter: 5´aaaaccggtttttatgggttttggtagg3´, 5´ttttctagaaagtatactatttattactcgatac3´; *mex-5* promoter: 5´aaatctagaatctgcaagaaaatacattttcg 3´, 5´tttaccggttctctgtctgaaacattcaa3´; *sysm-1* gene: 5´aaaaccggtaatgcagatttctgcattg3´, 5´tttaccggtatgcgttatctcatcggatt3´

### Brood size characterization

5 synchronized L4 worms for each strain was transferred to 5 separated plates and allowed egg laying for 3 days. Worms were transferred to fresh plates every day, in order to prevent starvation. Embryos were counted every day and the total number of embryos laid in three days were considered as brood size.

### Western blot analysis of phospho-PMK-1

To determine the phospho-PMK-1 level alteration upon DNA damage, 250 synchronized L4 worms from each genotype were transferred to a fresh OP50- seeded plate and were IR-treated with 0 or 90 Gy 20 h post-L4 at young adult stage. At the desired time points after IR treatment, worms were washed at least three times with M9 buffer to remove OP50 bacteria and were lysed directly in 70 μl of 2× SDS sample buffer. Samples were subsequently boiled for 5 min at 95 °C before being cooled down on ice for 10 min. Samples were sonicated two times for 20 s at 60% using a Bandelin Sonopuls sonicator prior to loading for electrophoresis. After completion of 10% Bis-Tris gel electrophoresis, the protein was transferred to a nitrocellulose membrane. The membrane was then blocked using 10% BSA in 1× TBS, containing 0.1% Tween 20 (TBST) for 1 h at room temperature. The protein membrane was probed overnight at 4 °C with rabbit phospho-p38 MAPK, Thr180/Tyr182 primary antibody (Cell Signaling TECHNOLOGY #9211), which was diluted in blocking buffer with a ratio of 1:1000. The membrane was next washed at least three times with 1× TBST and incubated with peroxidase-conjugated monoclonal mouse anti-rabbit secondary antibody (Jackson ImmunoResearch #211-032-171) with a dilution of 1:10,000 in blocking buffer for 1 h at room temperature. The protein membrane was then washed three times with 1× TBST prior to developing. As a loading control, the protein membrane was probed with mouse alpha-tubulin monoclonal antibody (SIGMA-ALDRICH #T6199) overnight at 4 °C with a dilution of 1:10,000 in 5% milk powder in 1× PBST followed by an incubation with peroxidase-conjugated goat anti-mouse secondary antibody (1:10,000) (Jackson ImmunoResearch #115-035-174) for 1 h at room temperature. The membrane was finally developed using the Amersham Enhanced Chemiluminescence (ECL) Prime Western Blotting Detection Reagent (#RPN2232).

### Immunofluorescence staining

Day 1 adult worms were picked from plates, and transferred to a drop of M9 buffer onto a 0.3% poly-lysine-treated three-well slide (3 × 14 mm printed wells slides from Fisher Scientific). Germline and intestine dissection was carried out with two syringe needles, followed by fixation with 3.7% formaldehyde for 1 h. Then a 24 × 24 mm coverslip was placed onto the drop, and the slide was left in −80 °C freezer for 10 min to perform the “freeze-cracking” procedure. Then the slide was quickly transferred to −20 °C methanol for <1 min. After fixation, slides were washed 1 time with 1× PBS and 2 times with 1× PBST. In order to improve the signal quality, slides were first blocked for 20 min with Image-iT FX signal enhancer (Thermo Fisher/Invitrogen, Carlsbad, CA) before blocking with 1× PBST containing 10% Donkey Serum for another 20 min. Afterward, primary antibodies diluted with 1× PBST containing 5% Donkey Serum were applied to the slides and incubated at 4 °C overnight. After 3 times washing with 1× PBST, the slides were incubated with secondary antibodies diluted with 1× PBST at 37 °C for 30 min. Then slides were washed with 1× PBST 3 times and mounted with DAPI Fluoromount-G mounting medium (Southern Biotech) and sealed with nail polish. Slides were stored at 4 °C in dark before imaging.

Primary antibodies used for immunofluorescence staining are Mouse monoclonal anti-V5 tag (SV5-P-K) antibody (Abcam, ab27671, dilution 1:50), Rabbit monoclonal anti-V5 tag (SV5-P-K) (Abcam, ab206566, dilution 1:50), rabbit phospho-p38 MAPK, Thr180/Tyr182 primary antibody (Cell Signaling TECHNOLOGY #9211, dilution 1:100) and mouse mAb SQV8 (Developmental Studies Hybridoma Bank) at 1 μg/ml. Secondary antibodies used are AlexaFluor 488 donkey anti-mouse IgG (Thermo Fisher/Invitrogen,Cat. No.: A21202, dilution 1:500 in PBS-T;) and AlexaFluor 594 donkey anti-rabbit IgG (Cat. No.: A21207, dilution 1:500 in PBS-T; Thermo Fisher/Invitrogen).

Fluorescence images for quantification were taken Zeiss Meta 710 confocal laser scanning microscope was used. For quantification, fixed exposure time was set for different treatments and strains. For p-PMK-1 staining, *Z*-stack images were taken with Zeiss Meta 710 confocal microscope, and the p-PMK-1 signal intensity was measured with the Imaris ×64 9.1.2 software. All of the fluorescence intensity was normalized to DAPI signal.

### qRT-PCR

For whole worm qRT-PCR, 200 synchronized L4 worms per genotype were transferred to three separate OP50-seeded plates to serve as three biological replicates. 20 h later and at young adult stage, worms were IR-treated with 0 and 90 Gy. Two hours after IR treatment, worms were washed with M9 buffer to remove excess of OP50 bacteria and were lysed in 1 ml TRIzol reagent (ThermoFisher Scientific #15596026), supplemented with 0.7 mm zirconia/silica beads (Roth #11079110z). The animals were further disrupted using Precellys24 (Bertin #P000669-PR240-A). 1-Bromo-3-chloropropane (SIGMA-ALDRICH #B9673) was used for phase separation prior to RNA isolation using RNeasy mini kit (Qiagen #74104). For RNA isolation from dissected germlines and intestines, synchronized day-1 adults were dissected with syringe needles in dissection buffer containing 1.1× egg salts buffer, 0.2% Tween 20 and 20 mM sodium azide. 25-30 germlines or intestines per condition were collected in 1 ml TRIzol reagent containing 0.7 mm zirconia/silica beads. To each sample, 20 μg of glycogen (Thermo Scientific) were added before tissue disruption by vortexing for 5 min and RNA isolation procedure. Reverse transcription into cDNA with 1 µg of isolated RNA was performed using Superscript II kit (Invitrogen #10328062). For qRT-PCR reaction, cDNA sample was mixed with SYBR Green I (SIGMA-ALDRICH #S9430) and Platinum Taq polymerase (Invitrogen #10966018). All qRT-PCR reactions were performed with 2 technical replicates. qRT-PCRs were normalized to *act-1*, *tbg-1* and *vha-6* housekeeping gene expression. For Normalization of qRT-PCR with dissected germlines, *syp-2* instead of vha-6 was used as housekeeping gene. For data analysis, the second derivative maximum method was applied, and induction of target cDNA was calculated according to the equation: (*E*_target_^ΔCP(cDNAref.-cDNAsample)target^)/(*E*_control_^ΔCP(cDNAref.-cDNAsample)control^). To calculate the relative expression of a target gene, each sample was compared to a reference sample, which is the un-treated WT. The following forward and reverse primers were used: *egl-1*: 5′tactcctcgtctcaggactt3′, 5′catcgaagtcatcgcacat 3′; *ced-13*: 5′ *gtcgtacaagcgtgatggat* 3′, 5′acggtgtttgagttgcaagc 3′; *sysm-1*: 5′ctgtaacgaagcagatgttagaagtg3′, 5′gggcattgttcagcaatattttcatc 3′; *sysm-1* targeting *ok3236* fragment: 5′catcggatttgtgattgtgct3′, 5′cgacaaccacttctaacatctg 3′.

### RNA in situ hybridization in dissected germline and intestine

The detection of *sysm-1* mRNA was carried out by a modified method described by Lee and Schedl^66^. Briefly, full-length *sysm-1* cDNA was amplified with forward primer 5′atgcgttatctcatcggatttg3′ and reverse primer 5′catgcagatttctgcattgg3′. The DIG-labeled single-strand DNA probes were generated with asymmetric (one-way) PCR, which gives anti-sense probes with reverse primer, and sense probes with forward primer.

Dissected intestines and germlines from each strain were transferred to 96-well plates with PBST. Fixation was performed with paraformaldehyde/glutaraldehyde fixation buffer for 2 h. Tissues were then digested with Proteinase K for 30 min and washed with PBST. Tissues were pre-hybridized in hybridization buffer (5× SSC, 50% deionized formamide, 100 μg/ml autoclaved Herring sperm DNA, 50 μg/ml Heparin and 0.1% Tween-20) for 1 h at 57 °C. Hybridization was performed at 57 °C for 36 h. Then tissues were washed with the following buffers: hybridization buffer at 57 °C, 50% hybridization buffer/50% PBST at 57 °C, PBST at 57 °C and PBST at room temperature. Tissues were then washed for 30 min in PBST prior to the detection.

Probes were detected with alkaline-phosphatase-mediated detection method. Briefly, alkaline-phosphatase-conjugated anti-DIG antibody (Roche (#1 093 274)) was diluted in PBST/BSA buffer, and incubated with tissues overnight at 4 °C. After three times washing with PBST/BSA, tissues were incubated with BCIP/NDT solution. Positive signal appeared at around 30 min–1 h after adding BCIP/NDT. The sense probe control gives a clean or background signal. The reaction was stopped by washing three times with PBST. Then the tissues were transferred to agar pad and mounted with the anti-fade solution. In situ hybridization images were taken with a bright field (Nomarski) microscope (ECLIPSE-Ci-E Microscope).

### Data presentation and statistical analysis

All the data and statistical significances were analyzed using the GraphPad Prism 7 software package (GraphPad). Statistical methods, sample size information and error bar descriptions are reported in the figure legends. Independent repeat experiments are shown in the supplementary section. Randomization was not applied because the group allocation was guided based on the genotype of the respective mutant worms. Worms of a given genotype were nevertheless randomly selected from large strain populations for each experiment without any preconditioning. Blinding was not applied as the experiments were carried out under highly standardized and predefined conditions such that an investigator-induced bias can be excluded. Averages of independent experiments are shown with mean ± s.e.m. When individual experiments, staining quantification, and male incidence as shown, median with 95% Cl was used as these data types contain outliers, which should not be shown as mean with SD. For the rest of experiments, such as qRT-PCT data, we use the common mean ± SD. The full statistical results are shown in Source data files.

### Reporting summary

Further information on research design is available in the Nature Research Reporting Summary linked to this article.

## Supplementary information

Supplementary Information

Reporting Summary

## Data Availability

All the independent experimental repeats for the graphs in this paper are available within the source data file of the article. Source data are provided with this paper.

## Source data

Source Data
