# Author Correction: Somatic PMK-1/p38 signaling links environmental stress to germ cell apoptosis and heritable euploidy

**DOI:** 10.1038/s41467-023-35831-7

**Published:** 2023-01-17

**Authors:** Najmeh Soltanmohammadi, Siyao Wang, Björn Schumacher

**Affiliations:** 1 Institute for Genome Stability in Ageing and Disease, Medical Faculty, University Hospital and University of Cologne, Joseph-Stelzmann-Str. 26, 50931 Cologne, Germany; 2 Cologne Excellence Cluster for Cellular Stress Responses in Ageing-Associated Diseases (CECAD), Center for Molecular Medicine Cologne (CMMC), University of Cologne, Joseph-Stelzmann-Str. 26, 50931 Cologne, Germany

**Keywords:** Apoptosis, Chromosomes, Genomic instability, Genomic instability, DNA damage and repair

Correction to: *Nature Communications* 10.1038/s41467-022-28225-8, published online 04 February 2022

The original version of this Article contained an error in Fig. 1c. The image shown in the bottom right panel was inadvertently duplicated from the top right panel. This error was introduced during the revision process. This has been corrected in both the PDF and HTML versions of the Article.

