# Supplementary material for: Somatic PMK-1/p38 signaling links environmental stress to germ cell apoptosis and heritable euploidy
Source: Nat Commun. 2022 Feb 4;13:701. doi: 10.1038/s41467-022-28225-8 (PMC8816960; doi:10.1038/s41467-022-28225-8)
Supplement: Supplementary file 1 — Supplementary Information [file 41467_2022_28225_MOESM1_ESM.pdf]

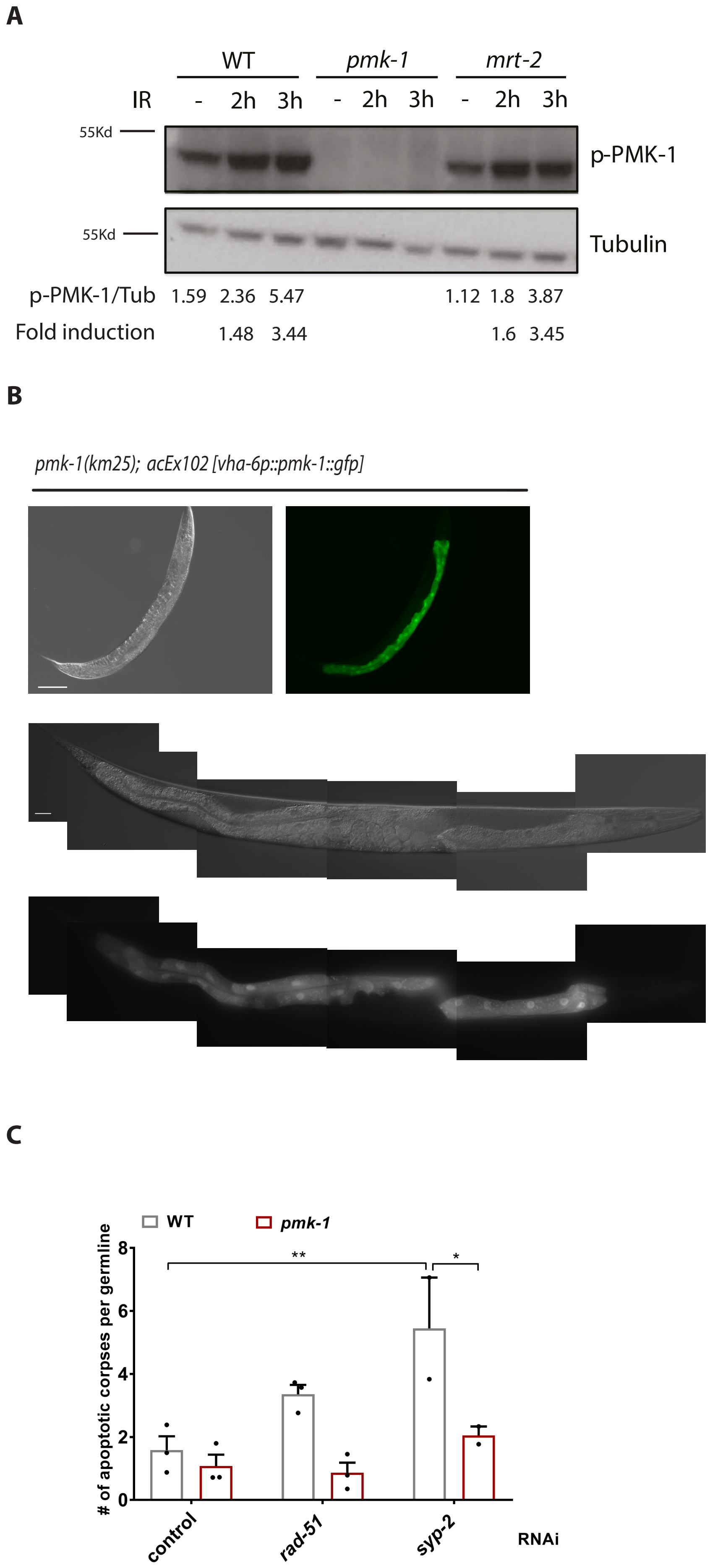

Soltanmohammadi et. al , Supplementary Figure 1.

Supplementary figure 1. Intestine-specific expression of *pmk-1* in *pmk-1(km25); acEx102 [vha-6p::pmk-1::gfp]* reporter line. (A) PMK-1 phophorylation is increased 2 to 3 hours after IR (90 Gy) comparable to WT worms. *mrt-2(e2663)* mutant also showed elevated level of PMK-1 phosphorylation after IR (90 Gy), indicating that activation of PMK-1 is not dependent on DDR checkpoint signaling pathways. 250 adult worms were lysed per each genotype, condition and time point for western blot analysis. Band densities were quantified using ImageJ. Each p-PMK-1 value was compared to its respective tubulin value; relative induction of p-PMK-1 upon IR treatment was calculated compared to non-treated condition of its respective genotype. Uncropped images are shown in the Source Data Files. This experiment was repeated three times with similar results, and the other two independent experiments are shown in Experimental repeats. **(B)** Representative DIC and fluorescent images of *pmk-1(km25); acEx102 [vha-6p::pmk-1::gfp]* reporter line indicative of intestine-specific expression of *pmk-1*. Upper images: the scale bar corresponds to 100 µm. Lower images: the scale bar corresponds to 20 µm. These experiments were repeated three times with similar observations. **(C)** Elevated baseline germ cell apoptosis in WT worms, mediated by *rad-51* and *syp-2* RNAi treatment is diminished in the *pmk-1(km25)* mutant animals. The graph summarizes n=3 independent experiments. Each experiment includes 10-20 germlines per condition scored 6h post-IR (90 Gy). Error bar shows mean ±sem (Two-way ANOVA with Tukey's multiple comparison test, *WT(control):syp-2(control) **p=0.0092*, *syp-2(control):syp-2(pmk-1) *p=0.0357*). Full statistical results and exact *p* values are shown in the Source Data Files.

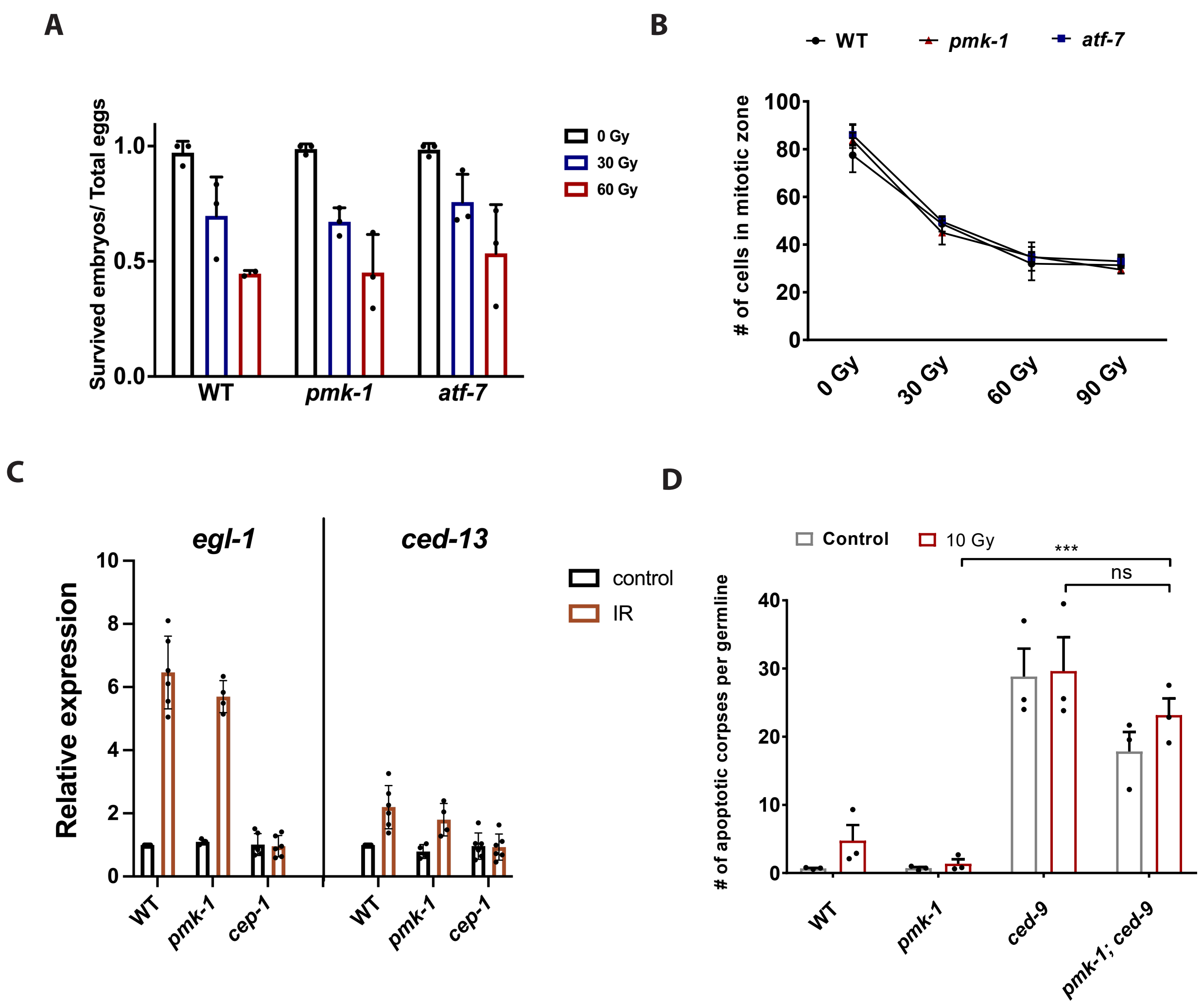

Soltanmohammadi et. al , Supplementary Figure 2.

Supplementary figure 2. PMK-1/p38 genetically acts downstream of CEP-1/p53 but upstream of the CED-9/Bcl-2 on the regulation of DNA damaged-induce germ cells apoptosis. (A) Embryonic survival in the F1 generation of IR-treated *pmk-1(km25)* and *atf-7(qd22,qd130)* mutant animals is comparable to that of WT worms, suggestive of no impairment in DNA repair mechanisms in these mutants. The graph shows the average of three biological replicates (Sample size: n= 3 adult worms used for 2 hours egg laying experiment). **(B)** Quantification of proliferative mitotic germ cells revealed that cell cycle arrest in response to IR damage is not affected by the *pmk-1(km25)* and *atf-7(qd22,qd130)* mutations. n=3 germlines per genotype and treatment were scored for the number of mitotic germ cells in the distal part of the germline. **(C)** The mRNA expression levels of *egl-1* and *ced-13* in *pmk-1(km25)* mutant phenocopied that of WT worms 2 hours after IR (90 Gy), suggesting CEP-1 transcription activity was not altered in *pmk-1(km25)* mutant worms. 200 adult worms were lysed for RNA isolation per each genotype and condition. qRT-PCR was performed using *egl-1* and *ced-13* primers. The qRT-PCR analysis includes n=3 biological and two technical replicates. Relative mRNA levels were compared to the WT untreated control and normalized to *act-1, tbg-1* and *vha-6* housekeeping gene expression. Error bars shown in **(A), (B),** and **(C)** is mean with SD. **(D)** CED-9 loss reverts the abrogated levels of apoptosis in *pmk-1(km25)* mutant at baseline as well as upon IR. Here, an IR dose of 10 Gy was used due to the high apoptosis levels in *ced-9* mutants. The graph summarizes n=3 independent experiments. Each experiment includes 10-20 germlines per condition scored 6h post-IR (90 Gy). Error bar shows mean ±sem (Two-way ANOVA with Tukey's multiple comparison test, *pmk-1(IR):pmk-1;ced-9(IR) ***p=0.0009, ced-9(IR):pmk-1;ced-9(IR) ns:p=0.7151*). Full statistical results and exact *p* values are shown in the Source Data Files.

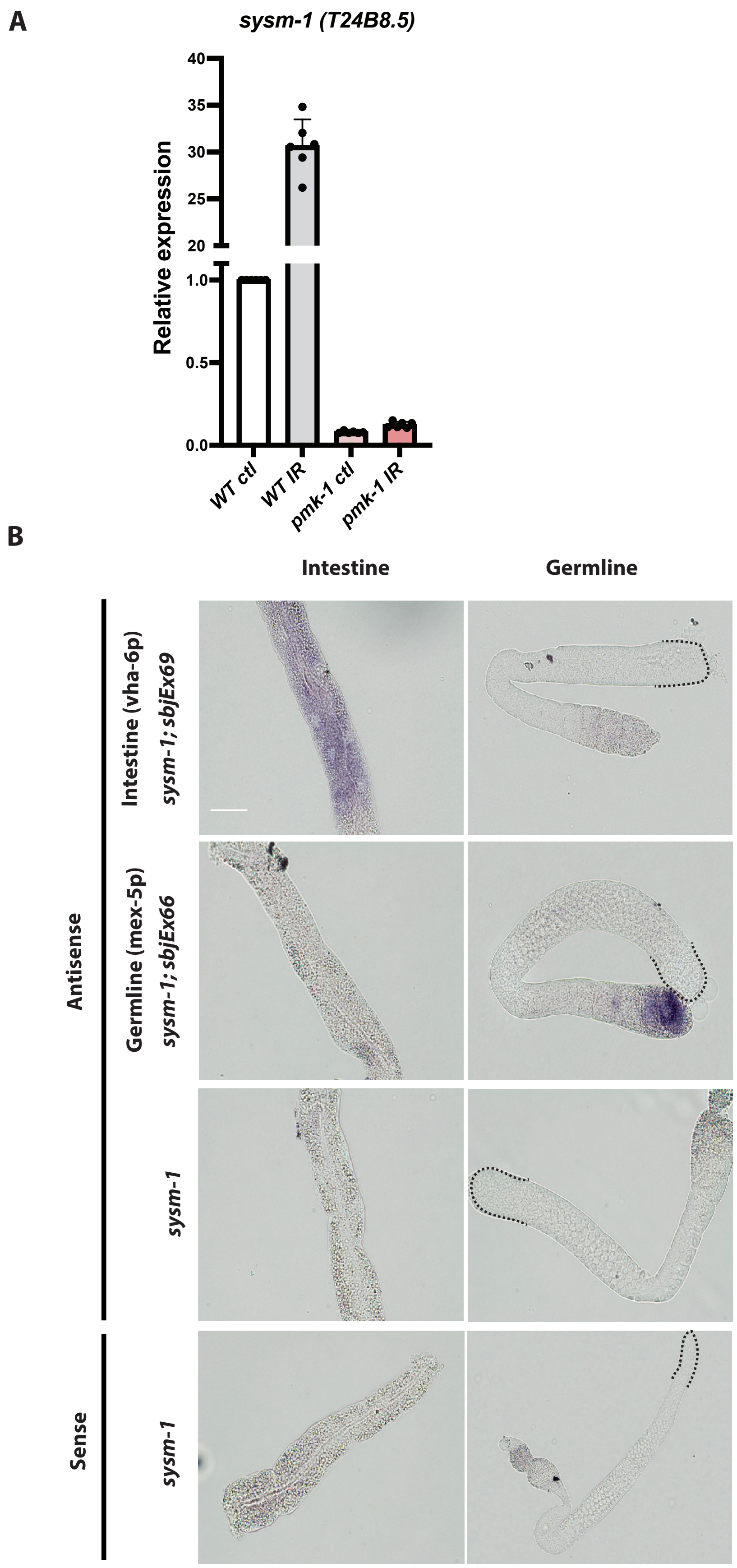

Soltanmohammadi et. al, Supplementary Figure 3.

Supplementary figure 3. ***sysm-1*** **mRNA upregulation in response to IR-induced DNA damage is PMK-1 dependent. (A)** The increased mRNA expression levels of *sysm-1* in WT animals are entirely suppressed in *pmk-1(km25)* mutant worms 2 h after IR treatment with 90 Gy, suggesting that *sysm-1* transcription in response to DNA damage is PMK-1 dependent. 200 adult worms were lysed for RNA isolation per genotype and condition. qRT-PCR was performed using *sysm-1* primers. The qRT-PCR analysis includes n=3 biological and two technical replicates. Relative mRNA levels were compared to the WT untreated control and normalized to *act-1, tbg-1* and *vha-6* housekeeping gene expression. Error bars show mean with SD. **(B)** Digoxigenin-labelled antisense DNA probes of *sysm-1* were hybridized to transcripts expressed in the dissected intestine (left panels) and germline (right panels) tissue. *sysm-1* mRNA was specifically detected in the intestine of *sysm-1; sbjEx69* (intestine specific transgene expression) and the pachytene region and more strongly in oocytes of the germline of *sysm-1; sbjEx66* (germline specific transgene expression) animals. *sysm-1* mRNA was not detected in the intestine and germline of *sysm-1* mutants. The sense probe control in *sysm-1* gives only background signal. Scale bar: 20µm. The dotted line was drawn for demarcation of the distal germline for orientation. This experiment was repeated three times with similar observations.

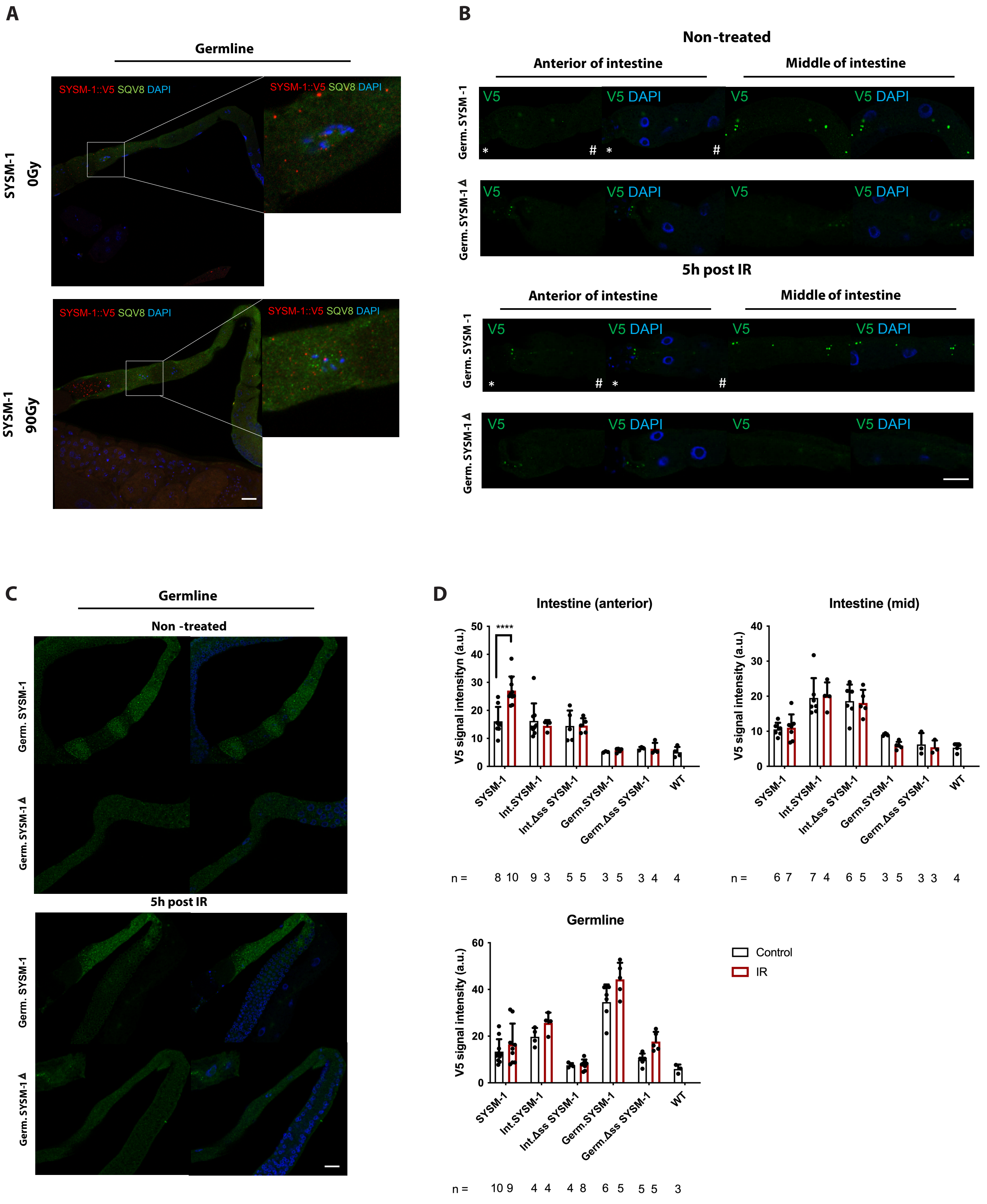

Soltanmohammadi et. al , Supplementary Figure 4.

Supplementary figure 4. Distribution pattern of germline promoter driven SYSM-1 expression. (A) Representative merged images from immunofluorescence co-staining of SYSM-1::V5 (red), the Golgi marker SQV8 (green) and DAPI in dissected germline with or without 90Gy IR. Zoomed-in images are shown at the right side, and no co-localized foci is observed. Scale bar= 20 μm. **(B)** Representative images of SYSM-1::V5 staining in dissected intestines of indicated strains with or without IR treatment are shown. * indicates anterior side; # indicates posterior side. Merged images with DAPI are placed at the right side. **(C)** Representative images of SYSM-1::V5 staining in dissected germlines of indicated strains with or without IR treatment are shown. Merged images with DAPI are shown at the right side. Scale bar= 20 μm. The experiments (**A**), (**B**), and (**C**) were repeated three times with similar observations. **(D)** Upper two panels show quantification of intestinal SYSM-1::V5 signal in Figure 3D and E. Lower panel shows the quantification of SYSM-1::V5 signal in Supplementary Figure 4B and C. The oocyte region of germline was used for quantification. Sample sizes in **D** are indicated. Error bars show mean with SD (Two-way ANOVA with Tukey's multiple comparison test, *SYSM-1(control):SYSM-1(IR) ****p<0.0001*).

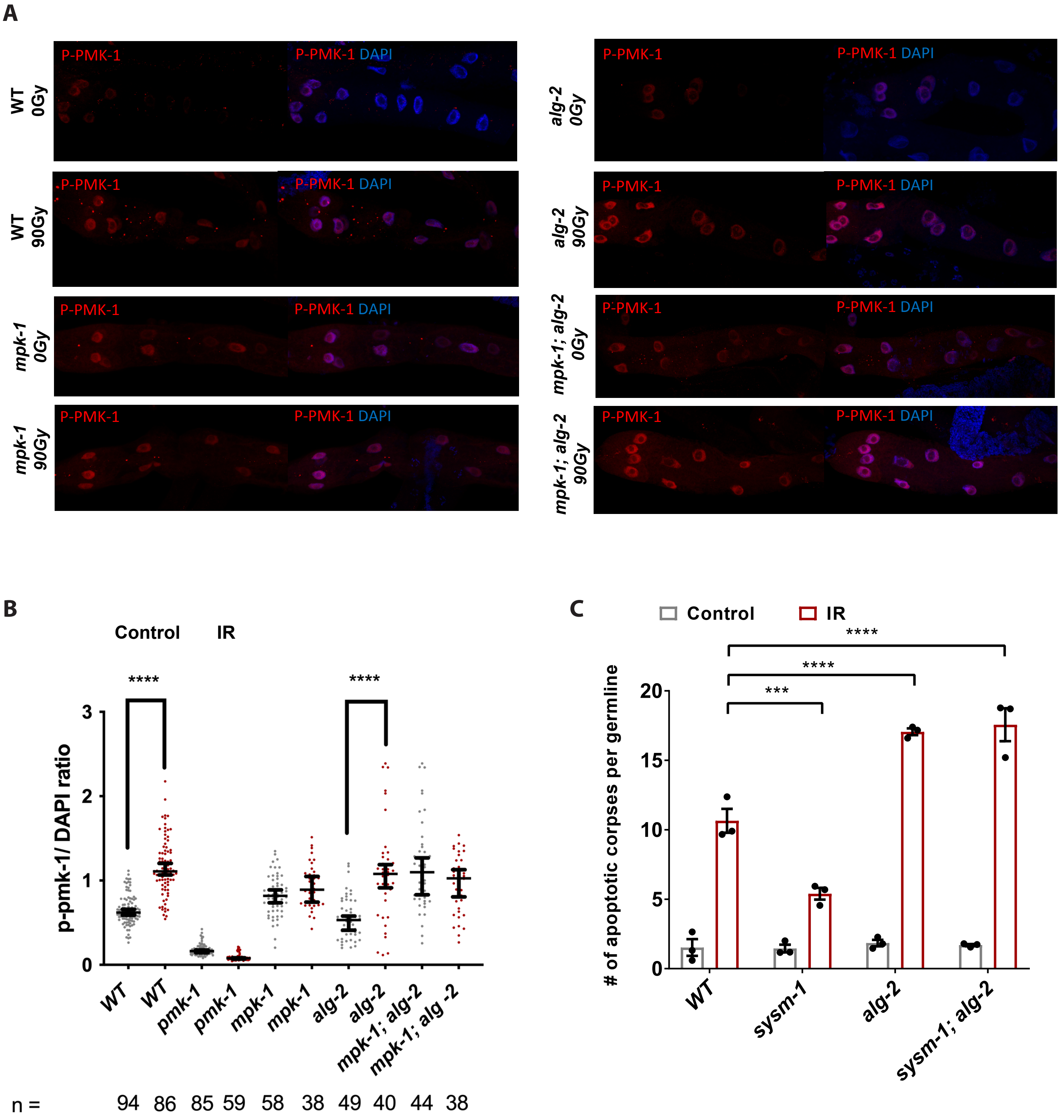

Soltanmohammadi et. al , Supplementary Figure 5.

Supplementary figure 5. p-PMK-1 staining in the *mpk-1*/ERK mutants. (A) p-PMK-1 staining of dissected intestines of indicated strains and treatment. Merged images with DAPI signal are shown. * indicates anterior side; # indicates posterior side. Scale bar: 20µm. This experiment was repeated three times with similar observations. (B) Quantification of p-PMK-1 signal intensity from A. DAPI was used for normalization. Sample sizes in **B** are indicated. One dot indicates relative quantification of one intestinal cell. Data are presented as median with 95% CI (One-way ANOVA test, *WT(control):WT(IR) ****p<0.0001, alg-2(control):alg-2(IR) ****p<0.0001,*). **(C)** Quantification of apoptotic corpses per germline of indicated strains with or without IR. The graph summarizes n=3 independent experiments, and each dot indicates one independent experiment. Each experiment includes 10-20 germlines per condition scored 6h post-IR (90 Gy). Error bar shows mean ±sem (Two-way ANOVA with Tukey's multiple comparison test, *WT(IR):sysm-1(IR) ***p=0.0003, WT(IR):alg-2(IR) ****p<0.0001, WT(IR):sysm-1;alg-2(IR) ****p<0.0001*). Full statistical results and exact *p* values are shown in the Source Data Files.

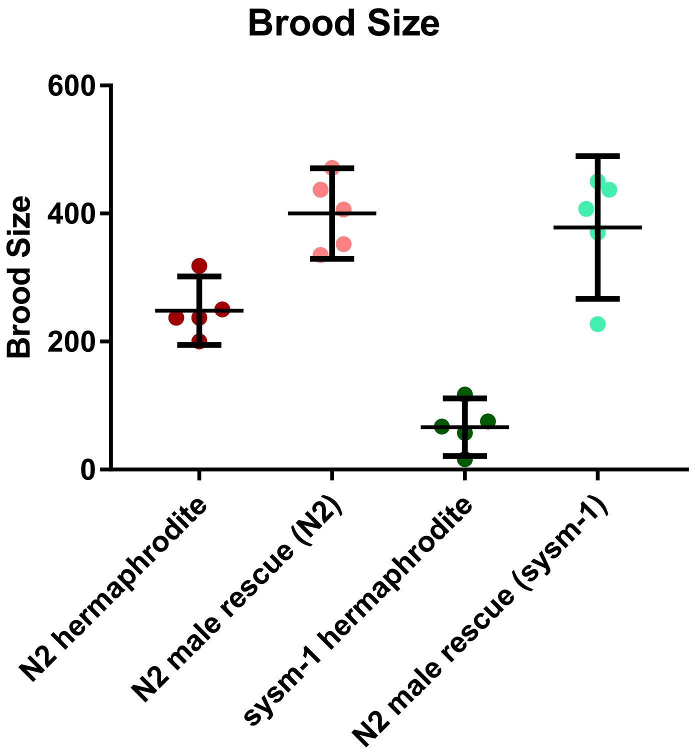

Soltanmohammadi et. al , Supplementary Figure 6.

Supplementary figure 6. Brood size of *sysm-1 (ok3236)*. Brood size of *sysm-1(ok3236)* is significantly lower compared to WT animals, and it can be completely rescued by mating with WT males, indicating a defect of spermatogenesis in *sysm-1(ok3236)* mutants; n= 5 animals for each group were analyzed in this experiment. Error bars show mean ±sd, Two-way ANOVA with Tukey's multiple comparison test was applied and full statistical results and exact *p* values are shown in the Source Data Files.

WT			
no heat shock		10 min 35°C heat shock	
progeny	male	progeny	male
73	0	65	0
93	0	45	0
78	0	79	0

pmk-1			
no heat shock		10 min 35°C heat shock	
progeny	male	progeny	male
77	0	82	0
75	0	64	0
65	0	67	0

Soltanmohammadi et. al , Supplementary Table 1.

Supplementary table 1. No meiotic failure upon short and acute exposure of worms to heat stress. Transient heat stress at 35°C for 10 min is not sufficient to elevate the male incidence in the F1 generation of WT and *pmk-1(km25)* mutant animals. The number of viable progeny is also not altered in WT and *pmk-1(km25)* mutants, while parental worms are exposed to short heat stress (Young adult worms heat shock treated at 35°C for 10 minutes). The table represents one experiment with three biological replicates (Sample size: n=3 adult worms used for 5 hours of egg laying experiment).
