# Supplementary material for: Somatic PMK-1/p38 signaling links environmental stress to germ cell apoptosis and heritable euploidy
Source: Nat Commun. 2022 Feb 4;13:701. doi: 10.1038/s41467-022-28225-8 (PMC8816960; doi:10.1038/s41467-022-28225-8)
Supplement: Supplementary file 2 — Reporting Summary [file 41467_2022_28225_MOESM2_ESM.pdf]

nature research

Corresponding author(s): Bjorn Schumacher

Last updated by author(s): Dec 3, 2019

Reporting Summary

Nature Research wishes to improve the reproducibility of the work that we publish. This form provides structure for consistency and transparency in reporting. For further information on Nature Research policies, see Authors & Referees and the Editorial Policy Checklist .

Statistics

For all statistical analyses, confirm that the following items are present in the figure legend, table legend, main text, or Methods section.

n/a	Confirmed	
☐	☒	The exact sample size (*n*) for each experimental group/condition, given as a discrete number and unit of measurement
☐	☒	A statement on whether measurements were taken from distinct samples or whether the same sample was measured repeatedly
☐	☒	The statistical test(s) used AND whether they are one- or two-sided<br>*Only common tests should be described solely by name; describe more complex techniques in the Methods section.*
☐	☒	A description of all covariates tested
☐	☒	A description of any assumptions or corrections, such as tests of normality and adjustment for multiple comparisons
☐	☒	A full description of the statistical parameters including central tendency (e.g. means) or other basic estimates (e.g. regression coefficient) AND variation (e.g. standard deviation) or associated estimates of uncertainty (e.g. confidence intervals)
☐	☒	For null hypothesis testing, the test statistic (e.g. *F*, *t*, *r*) with confidence intervals, effect sizes, degrees of freedom and *P* value noted<br>*Give P values as exact values whenever suitable.*
☒	☐	For Bayesian analysis, information on the choice of priors and Markov chain Monte Carlo settings
☒	☐	For hierarchical and complex designs, identification of the appropriate level for tests and full reporting of outcomes
☒	☐	Estimates of effect sizes (e.g. Cohen's *d*, Pearson's *r*), indicating how they were calculated

Our web collection on statistics for biologists contains articles on many of the points above.

Software and code

Policy information about availability of computer code

Data collection	Apoptotic and mitotic germ cells' characterization were counted using the Zeiss Axio Imager M1/2. Immunofluorescence images were taken by Zeiss Meta 710 confocal laser scanning microscope. In situ hybridization images were taken by ECLIPSE- Ci-E Microscope.
Data analysis	Characterization data were analyzed with GraphPad Prism 7 software package, and Immunofluorescence images were analyzed with Imaris x64 9.1.2 software.

For manuscripts utilizing custom algorithms or software that are central to the research but not yet described in published literature, software must be made available to editors/reviewers. We strongly encourage code deposition in a community repository (e.g. GitHub). See the Nature Research guidelines for submitting code & software for further information.

Data

Policy information about availability of data

All manuscripts must include a data availability statement. This statement should provide the following information, where applicable:

- Accession codes, unique identifiers, or web links for publicly available datasets
- A list of figures that have associated raw data
- A description of any restrictions on data availability

All the independent replicates for the graphs in this paper are available within the article and its Supplementary Figure, experimental repeats. Source data are provided as Source Data files.

Field-specific reporting

Please select the one below that is the best fit for your research. If you are not sure, read the appropriate sections before making your selection.

Life sciences study design

All studies must disclose on these points even when the disclosure is negative.

Sample size	Sample size was determined empirically or based on the similar data reported in other scientific publications.
Data exclusions	No data were excluded
Replication	All experiments were at least three times independently repeated as indicated in the figure legends.
Randomization	Randomization was not applied because the group allocation was guided based on the genotype of the respective mutant worms. Worms of a given genotype were nevertheless randomly selected from large strain populations for each experiment without any preconditioning.
Blinding	Blinding was not applied as the experiments were carried out under highly standardized and predefined conditions such that an investigator-induced bias can be excluded.

Reporting for specific materials, systems and methods

We require information from authors about some types of materials, experimental systems and methods used in many studies. Here, indicate whether each material, system or method listed is relevant to your study. If you are not sure if a list item applies to your research, read the appropriate section before selecting a response.

Materials & experimental systems

n/a	Involved in the study	
☐	☒	Antibodies
☒	☐	Eukaryotic cell lines
☒	☐	Palaeontology
☐	☒	Animals and other organisms
☒	☐	Human research participants
☒	☐	Clinical data

Methods

n/a	Involved in the study	
☒	☐	ChIP-seq
☒	☐	Flow cytometry
☒	☐	MRI-based neuroimaging

Antibodies

Antibodies used

Mouse monoclonal anti-V5 tag (SV5-P-K) antibody (Abcam, ab27671, dilution 1:50),
Rabbit monoclonal anti-V5 tag (SV5-P-K) (Abcam, ab206566, dilution 1:50),
Rabbit phospho-p38 MAPK, Thr180/Tyr182 primary antibody (Cell Signaling TECHNOLOGY #9211, dilution 1:100) and
Mouse mAb SQV8 (Developmental Studies Hybridoma Bank) at 1 µg/ml.
AlexaFluor 488 donkey anti-mouse IgG (Thermo Fisher/Invitrogen ,Cat. No.: A21202, dilution 1:500 in PBS-T;)
AlexaFluor 594 donkey anti-rabbit IgG (Cat. No.: A21207, dilution 1:500 in PBS-T; Thermo Fisher/Invitrogen).
Alkaline-phosphatase-conjugated anti-DIG antibody (Roche (#1 093 274))

Validation

Mouse monoclonal anti-V5 (SV5-P-K) tag (https://www.abcam.com/v5-tag-antibody-sv5-pk1-ab27671.html)
Rabbit monoclonal anti-V5 tag (SV5-P-K) (https://www.abcam.com/v5-tag-antibody-sv5-p-k-ab206566.html)
Anti-phospho-p38 MAPK, Thr180/Tyr182 (https://www.cellsignal.de/products/primary-antibodies/phospho-p38-mapk-thr180-tyr182-antibody/9211)
Anti- SQV8 (https://dshb.biology.uiowa.edu/SQV8)
anti-mouse IgG (https://www.thermofisher.com/antibody/product/Donkey-anti-Mouse-IgG-H-L-Highly-Cross-Adsorbed Secondary-Antibody-Polyclonal/A-21202)
anti-rabbit IgG (https://www.thermofisher.com/antibody/product/A-21207.html)
anti-DIG antibody (https://www.sigmaaldrich.com/DE/de/product/roche/11093274910)

Animals and other organisms

Policy information about studies involving animals; ARRIVE guidelines recommended for reporting animal research

Laboratory animals

Caenorhabditis elegans, hermaphrodites, day-1 adult. Strains used in this study are:
Strains used were N2 (Bristol; wildtype), KU25 pmk-1(km25), VC8 jnk-1(gk7), ZD318 atf-7(qd22,qd130), AV276 +/nT1[qIs51](IV); syp-2(ok307)/nT1[qIs51](V), BJS717 syp-2(ok307) V/nT1[qIs51](IV); pmk-1(km25)IV, AY102 pmk-1(km25) IV; acEx102 [vha-6p::pmk-1::GFP + rol-6(su1006)], CB5348 mrt-2(e2663), XY1054 cep-1(lg12501), PP935 ced-9(n1653), BJS373 pmk-1(km25); ced-9(n1653), VC2477 sysm-1 (ok3236), SD939 mpk-1(ga111) unc-79(e1068); WM53 alg-2 (ok304); BJS710 sysm-1(ok3236); sbjEX65 [sysm-1p::SYSM-1::GFP + myo-2::tdTomato], BJS751 sysm-1(ok3236); sbjEx66 [mex-5p::SYSM-1::GFP + myo2::dtTomato], BJS818 sysm-1 (ok3236); sbjEx69 [vha-6p::SYSM-1::GFP + myo-2::tdTomato], BJS844 syp-2(ok307) V/nT1 [qIs51](IV); pmk-1(km25)IV; acEx102 [vha-6p::pmk-1::GFP + rol-6(su1006)]; BJS984 pmk-1(km25); sysm-1 (ok3236); sbjEx69

	[vha-6p::SYSM-1::GFP + myo-2::tdTomato]; BJS985 sysm-1 (ok3236); alg-2 (ok304); BJS608 alg-2(ok304);mpk-1(ga111) unc-79 (e1068). 5 strains were generated by SunyBiotech (China) and verified with genotyping PCR, they are: PHX3100 sysm-1(syb3100 [vha-6p::sysm-1::V5]); PHX3078 sysm-1(syb3078[mex-5p::sysm-1::V5]); PHX3073 sysm-1(syb3073[vha-6p::sysm-1(Δss)::V5]); PHX 3060 sysm-1(syb3060[mex-5p::sysm-1(Δss)::V5]); PHX2848 sysm-1(syb2848[sysm-1::V5]).
Wild animals	The study did not involve wild animals.
Field-collected samples	The study did not involve samples collected in the field
Ethics oversight	No ethical approval was required because C. elegans is a non-vertebrate species.

Note that full information on the approval of the study protocol must also be provided in the manuscript.
